# Involvement of Potato (*Solanum tuberosum* L.) MKK6 in Response to *Potato virus Y*

**DOI:** 10.1371/journal.pone.0104553

**Published:** 2014-08-11

**Authors:** Ana Lazar, Anna Coll, David Dobnik, Špela Baebler, Apolonija Bedina-Zavec, Jana Žel, Kristina Gruden

**Affiliations:** 1 Department of Biotechnology and Systems Biology, National Institute of Biology, Ljubljana, Slovenia; 2 Laboratory for Molecular Biology and Nanobiotechnology, National Institute of Chemistry, Ljubljana, Slovenia; Instituto de Biología Molecular y Celular de Plantas, Spain

## Abstract

Mitogen-activated protein kinase (MAPK) cascades have crucial roles in the regulation of plant development and in plant responses to stress. Plant recognition of pathogen-associated molecular patterns or pathogen-derived effector proteins has been shown to trigger activation of several MAPKs. This then controls defence responses, including synthesis and/or signalling of defence hormones and activation of defence related genes. The MAPK cascade genes are highly complex and interconnected, and thus the precise signalling mechanisms in specific plant–pathogen interactions are still not known. Here we investigated the MAPK signalling network involved in immune responses of potato (*Solanum tuberosum* L.) to *Potato virus Y*, an important potato pathogen worldwide. Sequence analysis was performed to identify the complete MAPK kinase (MKK) family in potato, and to identify those regulated in the hypersensitive resistance response to *Potato virus Y* infection. *Arabidopsis* has 10 MKK family members, of which we identified five in potato and tomato (*Solanum lycopersicum* L.), and eight in *Nicotiana benthamiana*. Among these, St*MKK6* is the most strongly regulated gene in response to *Potato virus Y*. The salicylic acid treatment revealed that St*MKK6* is regulated by the hormone that is in agreement with the salicylic acid-regulated domains found in the St*MKK6* promoter. The involvement of St*MKK6* in potato defence response was confirmed by localisation studies, where StMKK6 accumulated strongly only in *Potato-virus-Y*-infected plants, and predominantly in the cell nucleus. Using a yeast two-hybrid method, we identified three StMKK6 targets downstream in the MAPK cascade: StMAPK4_2, StMAPK6 and StMAPK13. These data together provide further insight into the StMKK6 signalling module and its involvement in plant defence.

## Introduction

Mitogen-activated protein kinase (MAPK) cascades are conserved signalling modules in eukaryotes that transduce extracellular stimuli downstream from the receptors, thus mediating the intracellular responses. The plant MAPK cascades have pivotal roles in the regulation of plant development and in responses to a variety of stress stimuli, including pathogen infection, wounding, temperature, drought, salinity, osmolarity, UV irradiation, ozone and reactive oxygen species [1].

In a general model of the MAPK signalling cascade, activation of plasma membrane receptors activates the MAPK kinase kinases (MKKKs). These are serine or threonine kinases that phosphorylate a conserved S/T-X3−5-S/T motif of the downstream MAPK kinases (MKKs), which, in turn, phosphorylate MAPKs on threonine and tyrosine residues in a conserved T-X-Y motif of their activation loop [2]. Following this MKK alteration of the phosphorylation-dependent properties of their target proteins, the activated MAPKs translate the information further, which eventually leads to changes in, e.g., gene expression, cellular redox state, or cell integrity [3].

The genome of the model plant *Arabidopsis thaliana* encodes 60 MKKKs, 10 MKKs and 20 MAPKs [4]. This indicates that the MAPK cascade might not simply consist of a single MKKK, MKK and MAPK connected together, but has the potential to be organised into many thousands of distinct MKKK–MKK–MAPK combinations, with some level of redundancy. To minimise unwanted cross-talk, the spatial and temporal activities of the different components must be strictly regulated [5].

Despite the potential multiplicity of MAPK cascades, only a small number of MAPK modules have been experimentally defined [6]. As the MKK family consists of a relatively small number of genes, their activity in different MAPK modules is widely dispersed [5]. In *Arabidopsis*, MKKs can be divided into four different groups (A–D) based on their sequence similarities [4], [7]. Group A includes *Arabidopsis thaliana* AtMKK1, AtMKK2 and AtMKK6. AtMKK1 and AtMKK2 act upstream of AtMAPK4 in response to cold, salinity and pathogens [8], [9]. AtMKK6 is involved in cytokinesis control and cell-cycle regulation [10]. The group B MKKs includes AtMKK3, which functions upstream of AtMAPK6 in the regulation of jasmonic acid (JA) signal transduction [11] and is involved in pathogen defence [12]; overexpression of AtMKK3 leads to enhanced tolerance to salt and increased sensitivity to abscisic acid [13]. The group C MKKs include AtMKK4 and AtMKK5, which act upstream of AtMAPK3 and AtMAPK6 in the regulation of plant development and defence responses [1], [14]–[16]. The group D MKKs include AtMKK7, AtMKK8, AtMKK9 and AtMKK10. AtMKK9 is involved in ethylene signalling [17] and in leaf senescence [18], while AtMKK7 is involved in plant basal and systemic resistance [19].

During pathogen attack, MAPK signalling is an indispensable component of the host defence response, in a way that it is involved in the crosstalk between secondary messengers and hormones [20]. The key hormones in plant biotic interactions include the salicylates, jasmonates and ethylene [21], whereby their specific roles depend on the particular host–pathogen interaction. Many studies have indicated that salicylic acid (SA) is a key regulatory compound in disease resistance against fungi, bacteria and viruses (reviewed in [22]). The importance of SA in viral multiplication and symptom development has also been confirmed in potato (*Solanum tuberosum* L.) - *Potato virus Y* (PVY) interaction [23], [24]. Depending on the virus, SA can induce inhibition of viral replication and cell-to-cell or long-distance viral movement (reviewed in [25]) and in agreement with this, SA is also a key component in the directing of events during and following hypersensitive resistance (HR) [21], [24]. Hypersensitive resistance is an efficient defence strategy in plants, as it restricts pathogen growth and can be activated during host, as well as non-host, interactions. It involves programmed cell death and manifests in necrotic lesions at the site of pathogen attack (reviewed in [26]). *Potato virus Y* is a member of the *Potyviridae* family and it is an important potato pathogen worldwide. In potato, HR is conferred by the *Ny* genes (reviewed in [27]). The potato cultivar (cv.) Rywal carries the *Ny-1* gene and it develops HR that is manifested as necrotic lesions on leaves 3 days following their inoculation with various PVY strains [28].

To date, a large number of members of the MAPK cascades from different species have been investigated, although to the best of our knowledge, there has been no systematic investigation of the MKK family and its function in defence signalling in potato. Moreover, no MAPK immune response network module has been defined for potato – PVY interactions.

We thus first performed sequence analysis of the complete MKK gene family in potato, and of its close relatives, where their genomes have been sequenced. Based on the present transcriptome data, StMKK6 was identified as the most responsive member after viral attack. We further investigated the role of StMKK6 in the response to PVY infection at the gene expression level, and studied its intracellular localisation and identified its downstream targets in the MAPK signalling cascade.

## Materials and Methods

### Bioinformatics analysis

*Arabidopsis thaliana MKK* gene family (The Arabidopsis Information Resource; TAIR: http://www.arabidopsis.org/) was blasted (tBLASTx algorithm) [29] against the potato [30], tomato (*Solanum lycopersicum* L.) and *Nicotiana benthamiana* (The SOL Genomics Network: http://solgenomics.net/) genomes to identify the *MKK* gene family in all three *Solanaceae* species. The names of the potato *MKK* genes were assigned based on the names of their apparent *A. thaliana* orthologues.

To identify and gather all of the available sequence information on St*MKK6* (GenBank accession number KF837127), St*MAPK4_1* (GenBank accession number KJ027594), St*MAPK4_2* (GenBank accession number KJ027595), St*MAPK6* (GenBank accession number KJ027596) and St*MAPK13* (GenBank accession number KJ027597), several database searches were performed. Sequences of our isolated genes were blasted (tBLASTx; [29]) against NCBI (National Centre for Biotechnology Information: http://www.ncbi.nlm.nih.gov/); UniProt (Universal Protein Resource: http://www.uniprot.org/); TAIR; PlantGDB (http://www.plantgdb.org/); POCI (Potato Oligo Chip Initiative: http://pgrc-35.ipk-gatersleben.de/pls/htmldb_pgrc/f?p=194∶1); The Gene Index Project (http://compbio.dfci.harvard.edu/tgi/); and TIGR Plant Transcripts Assemblies (http://plantta.jcvi.org/search.shtml).

To identify St*MKK6* orthologues we searched the UniProt database using the BLASTp algorithm [29]. Multiple-sequence alignments were performed using the MAFFT programme (version 7) [31]. Phylogenetic trees were constructed based on protein sequences using the MEGA 5 software [32], with the neighbour-joining method [33]. Bootstrap values were derived from 1,000 replicates, to quantify the relative support for branches of the inferred phylogenetic tree. To distinguish between *MKK6* orthologues and other *MKK*s, other At*MKK*s were included in the first MAFFT analysis.

Information on the targets of AtMKK6 was collected from the *Arabidopsis* Interactome Network Map [34]. The corresponding targets in potato were assigned by *A. thaliana*-potato orthologue and potato paralogue connections [35].

The location of the St*MKK6* gene (PGSC0003DMG403005720) within the potato genome was identified using the Ensembl Plants portal (http://plants.ensembl.org/index.html). The promoter sequences were obtained from the *Solanum_tuberosum*-PGSC_DM_v34_superscaffolds database. Promoter sequences were analysed using the PlantCARE web service [36].

Localisation predictions of the *A. thaliana* and potato MKK6 proteins were performed using PredictProtein [37] and the data on the whole *A. thaliana* MKK family using the SUBA3 web services [38].

### Metadata analysis

The expression data of the St*MKK* genes in developmental tissues and under stress conditions were collected through the use of the Bio-Analytic Resource for Plant Biology (http://bar.utoronto.ca/welcome.htm) and the Potato eFP browser [39]. The developmental tissues were vegetative and reproductive organs from greenhouse-grown plants. For each *MKK*, the RNA expression signals (FPKM values) based on the RNA sequencing of double monoploid *S. tuberosum* Group Phureja clone DM1-3 (DM) [40] and the genome sequence and analysis of the tuber crop potato [30] were collected. At*MKK6* transcription data from various datasets were analysed using Genevestigator [41] and only two-fold changes were regarded as significant.

We collected data from previously reported microarray expression analyses of the potato HR cv. Rywal and the SA deficient NahG-Rywal infected with PVY^N-Wi^ ([24], GEO: GSE46180), for all of the potato *MKK*s and *MAPK*s. Microarray identifiers were connected to the PGSC gene identifiers [35] and only the probes with less than four single nucleotide polymorphisms compared to the PGSC transcripts were used.

Co-expression analysis of microarray expression datasets was carried out with Biolayout Express3D 3.0 software [42], with ratios of PVY/mock normalised signals. The network was constructed using the Pearson correlation coefficient threshold of 0.98, and the graph was clustered using the Markov clustering algorithm with inflation 2.3; the other parameters were set at the default values.

### Gene expression analysis

Total RNA from control and SA- treated samples was extracted using MagMAX-96 Total RNA Isolation Kit (Life Technologies) according to manufacturer's instructions. RNA concentration was quantified by UV absorption at 260 nm using a NanoDrop ND1000 spectrophotometer (Nanodrop technologies). Integrity and purity of the RNA samples were determined by agarose gel electrophoresis and OD 260/280 nm absorption ratios. To confirm microarray results the same RNA samples previously analysed in microarray experiments [24] were used. Reverse transcription was performed on 1 µg of total RNA using High Capacity cDNA Reverse Transcription Kit (Applied Biosystems). The expression of St*MKK6* was assayed by real-time PCR (qPCR). The primers and probe (MKK6_F, MKK6_R and MKK6_S) targeting St*MKK6* were designed by Primer Express 2.0 (Applied Biosystems) (Table S1 in File S1). The qPCR was performed using TaqMan chemistry, with Cq values determined as described previously [24]. All reactions were run on a 7500 Fast Real-Time PCR System (Applied Biosystems) in 5 µl volume with 300 nM concentration of primers and 150 nM of probe and performed in duplicate. Linearity (R2) and efficiency (E = 10[−1/slope]) [43] of each reaction were compared to the accepted values. The standard curve method was used for relative gene expression quantification. Target gene accumulation was normalised to two endogenous control genes, cytochrome oxidase (COX) [44] and elongation factor 1 (EF-1) [45]. To determine differentially expressed genes, the Student t-test was performed.

### Cloning

The full-length sequences of St*MKK6*, St*MAPK4_1*, St*MAPK4_2*, St*MAPK6* and St*MAPK13* were amplified from potato cv. Rywal cDNA with the primers listed in Table S1 in File S1. The fragments were inserted into the pJET 1.2 blunt cloning vector (Thermo Scientific) and sequenced (GATC Biotech). For the localisation studies, St*MKK6* was cloned into the pENTR/D TOPO vector (Invitrogen), using the LR reaction, following the manufacturer protocol, and recombined into the binary destination vectors pH7YWG2 (YFP) and PH7CWG2 (CFP) [46], to produce proteins with C-terminal YFP/CFP fusion.

The St*MKK6* promoter was amplified using the genomic library from potato cv. Santé as the template, which was constructed using GenomeWalker Universal kit (BD Biosciences Clontech), as described previously [47]. In the genome walking PCR amplifications, the Advantage 2 Polymerase Mix (Clontech) was used with the PCR conditions suggested by the manufacturer. The adaptor primer AP1 and a nested primer AP2, that were provided by the manufacturer were paired with the nested reverse gene-specific primers MKK6_AP1 and MKK6_AP2 for amplification of the region upstream from the St*MKK6* gene. The fragment was inserted into the pJET 1.2 blunt cloning vector (Thermo Scientific) and sequenced. The 200-bp region of the St*MKK6* promoter (identical in all three of the investigated genotypes) was afterwards cloned in front of the St*MKK6* gene in the pH7YWG2 vector, using QuikChange II XL Site-Directed Mutagenesis kit (Agilent Technologies), with the MAPKprom-YFP_F and MKK6prom-YFP_R primers. The St*MKK6* promoter region from potato cv. Rywal was amplified from the genomic DNA using the primers listed in Table S1 in File S1.

For the yeast two-hybrid analysis, the coding regions of the selected genes were cloned into pGBKT7 (St*MKK6*) or pGADT7 (St*MAPK4_1*, *- 4_2*, *- 6*, *-13*) using QuikChange II Site-Directed Mutagenesis kit (Agilent Technologies). The primers used are listed in Table S1 in File S1.

### Hormonal treatment

Two different potato genotypes were used in this study including 1 non-transgenic cv. Rywal and 1 transgenic line NahG, transgenic plants of cv. Rywal (NahG-Rywal) [24]. Potato plants were grown in stem node tissue culture. Two weeks after node segmentation they were transferred to soil and grown as previously described by Baebler et al. 2009 [45].

SA treatment was performed spraying plants with SA-analog 300 µM INA (98% 2,6-Dichloroisonicotinic acid, Aldrich) in distilled water. Control plants were sprayed with distilled water alone. Leaves were harvested after 24 h of treatment and immediately frozen in liquid nitrogen. Two biological replicates per treatment were analysed.

### Localisation studies

*N. benthamiana* plants were grown in a growth chamber at 22/20°C under a 16/8 h light/dark cycle. PVY^NTN^ (isolate NIB-NTN, AJ585342) inoculation of *N. benthamiana* was performed as described by Baebler et al. 2009 [45]. Plasmids pH7YWG2 (YFP), pH7CWG2 (CFP), pH7YWG2::StMKK6-YFP, pH7CWG2::StMKK6-CFP under the 35S promoter, and pH7YWG2::StMKK6 under the control of the native promoter, were introduced into *Agrobacterium tumefaciens* strain GV3101 by electroporation. The *A. tumefaciens* cells were cultured, harvested by centrifugation, and re-suspended in 0.2 mM acetosyringone solution, and infiltrated into 4–5-week-old *N. benthamiana* leaves, 8 days after PVY/mock inoculation.

After 48 h to 72 h, YFP/CFP were visualized with a Leica TCS SP5 laser-scanning microscope mounted on a Leica DMI 6000 CS inverted microscope (Leica Microsystems, Germany), with an HC PL FLUOTAR 10.0×0.30 DRY objective. For excitation, the 458 nm (enhanced cyan fluorescent protein) and 514 nm (enhanced yellow fluorescent protein) lines of an Argon laser were used. The enhanced cyan fluorescent protein emission was measured from 475 nm to 495 nm, and the enhanced yellow fluorescent protein from 525 nm to 550 nm. Autofluorescence emission was measured from 690 nm to 750 nm. Differential interference contrast (DIC) images were captured using the transmission light detector of the confocal microscope. All of the images were acquired with the 10× objective or with an additional 3.64× zoom. The acquired images were processed using the Leica LAS AF Lite software (Leica Microsystems). pH7YWG2 and pH7CWG2 without the inserted St*MKK6* were used as the controls.

### Yeast two-hybrid assay

The coding regions of selected genes were cloned into pGBKT7 (St*MKK6*) or pGADT7 (St*MAPK4_1*, *-4_2*, *-6*, *-13*). To introduce insertions into plasmids the QuikChange II Site-Directed Mutagenesis Kit (AgilentTechnologies) was used. The primers used are listed in Table S1 in File S1.

Yeast two-hybrid analysis was performed using the Matchmaker GAL4-based two-hybrid assay (Clontech). The vector pGBKT7, containing bait protein fused to Gal DNA-binding domain, was used to transform the yeast strain Y2H Gold through the polyethylene glycol/LiAc-based method (Clontech), according to manufacturer instructions. After selection on SD/-Leu medium, the transformed colonies were used for co-transformation following the protocol described by Clontech, with the pGADT7 vector. It was used as a prey and contained target genes fused to Gal DNA-activation domain. Co-transformants were selected on SD/-Leu/-Trp (DDO) medium. Positive interaction transformants were selected on SD/-Leu/-Trp/-His/-Ade/x-a-Gal/Aba (QDO/X/A) medium. All of the genes were previously tested for autoactivation and toxicity, plating yeast colonies transformed with pGBKT7_StMKK6 on SD/-Trp/x-a-Gal/Aba and pGADT7_StMAPK4_1, *-*4_2, *-*6, *-*13 on SD/-Leu/x-a-Gal/Aba. Positive (pGBKT7_SV40 large T-antigen -pGADT7_53) and negative (pGBKT7_SV40 large T-antigen -pGADT7_Lam) interaction controls, included in the Matchmaker Gold Yeast Two-Hybrid System (Clontech), were performed in parallel.

## Results

### The *MKK* family in potato

**Figure 1:**
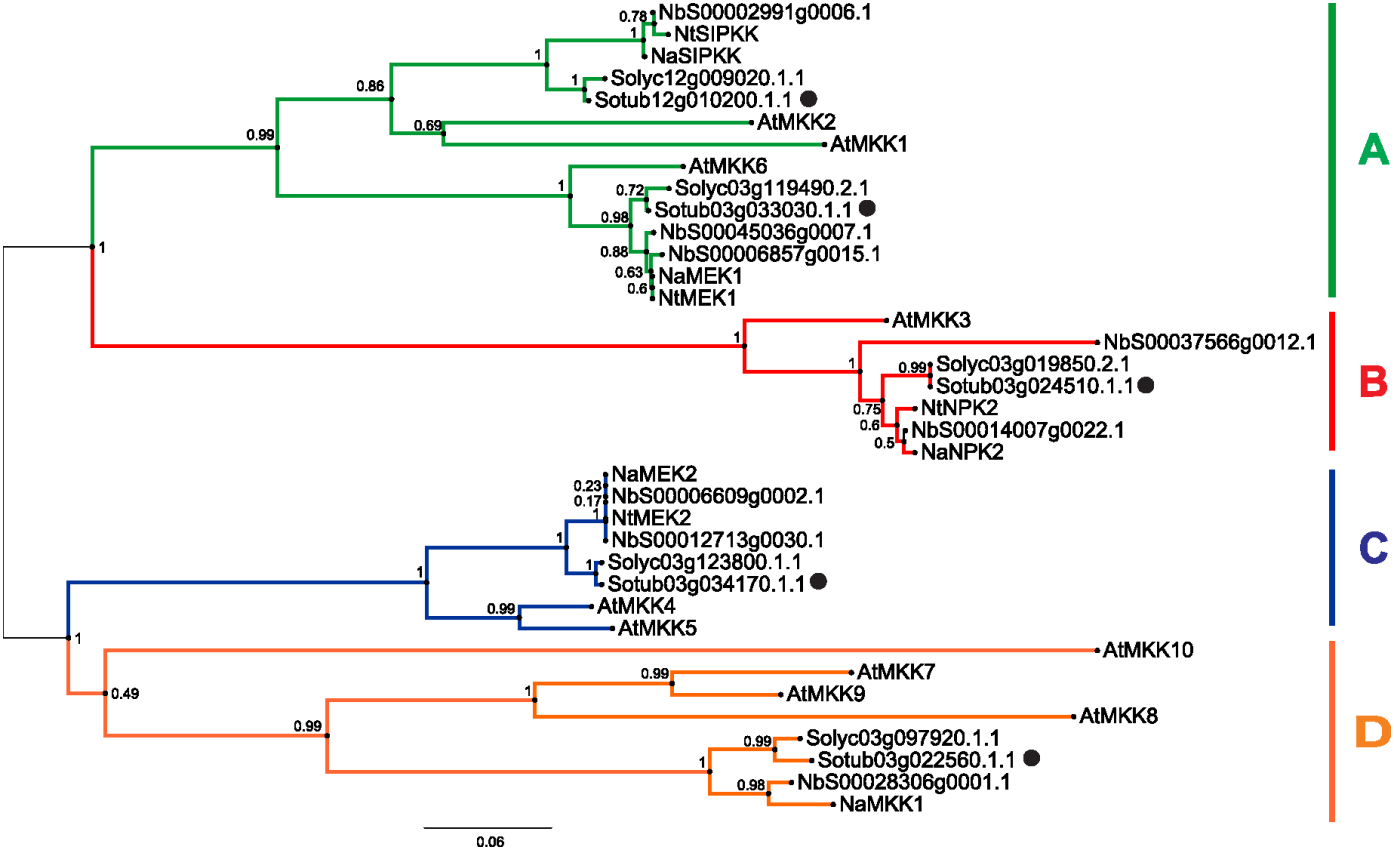
Phylogenetic tree of *MKK* family in *A. thaliana* and five *Solanaceae* species. The species are *A. thaliana* (At), potato (Sotub), tomato (Solyc), *N. benthamiana* (Nb), *N. attenuata* (Na) and *N. tabacum* (Nt). Genes are grouped into 4 groups: A (green), B (red), C (blue) and D (orange) [4]. Potato genes are marked with dots. The numbers on the nodes are percentages from a bootstrap analysis of 1000 replicates. The scale bar indicates the branch length that corresponds to 0.06 substitutions per site.

Our genome analysis of the potato, tomato and *Nicotiana attenuata* genomes identified five *MKK* genes in each, with four in the tobacco *Nicotiana tabacum* L. genome, and eight in the *N. benthamiana* genome (Figure 1). Based on the phylogenetic tree of *Arabidopsis* and five *Solanaceae* species, for only two of the *A. thaliana* genes (At*MKK3*, At*MKK6*) a specific orthologue could be assigned from potato, tomato or *Nicotiana* spp., while the other *MKK*s form larger orthologue groups. The *MKK* family in potato, tomato and *Nicotiana* spp. can also be divided into four groups: group A (*A. thaliana* and *N. benthamiana*, three genes; potato, tomato, *N. tabacum* and *N. attenuata*, two genes); B (*N. benthamiana*, two genes; the rest, one gene each); C (*A. thaliana* and *N. benthamiana*, two genes; potato, tomato, *N. tabacum* and *N. attenuata*, one gene each); and D (*A. thaliana*, four genes; *Solanaceae* spp., one gene each, except *N. tabacum* which has none). Out of the five examined *Solanaceae* spp., *N. benthamiana* is the only one of these in which duplication of the *MKK* genes occurred after speciation and thus *N. benthamiana* has possible paralogues for *MKK3* (NbS00014007g0022.1, NbS00037566g0012.1), *MKK4/5* (NbS00012713g0030.1, NbS00006609g0002.1) and *MKK6* (NbS00006857g0015.1, NbS00045036g0007.1) (Figure 1).

### Regulation of potato *MKK* family gene expression in developmental processes and stress

We first analysed the gene-expression metadata for the potato *MKK* family for developmental processes and after exposure to different biotic and abiotic stress [40]. The tissue specificities of the *MKK* family members differed between both of the potato varieties studied here, the potato Group Phureja Clone DM, and Group Tuberosum Clone RH, especially regarding the expression in tubers and stolons. In general, this analysis showed that in potato, St*MKK1/2* and St*MKK4/5* are relatively uniformly expressed, with the highest expression in tubers and stolons. St*MKK3* transcripts had the highest abundance in carpels and petals, and St*MKK7/9* in tubers and flowers, and also in roots for Clone RH. Under biotic and abiotic stress conditions, all of the potato *MKK*s except St*MKK4/5*, were down-regulated after wounding. None of the other stress conditions and hormonal treatments influenced the *MKK* family gene expression by more than two-fold compared to controls (File S2).

**Figure 2:**
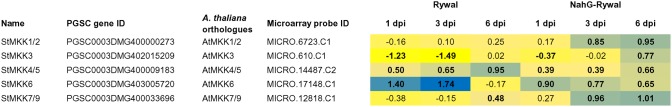
Gene expression pattern of *MKK* family in the HR response against PVY. Cultivar Rywal (HR response, conferred by *Ny-1* gene) and NahG-Rywal (impaired accumulation of SA) were analysed for whole transcriptome response 1, 3 and 6 days after PVY^N-Wi^ infection [24]. *A. thaliana* and *S. tuberosum* PGSC orthologues were assigned to each probe. Log_2_ fold changes of PVY in infected vs. mock-inoculated plants are indicated for each time point (1, 3 and 6 dpi). Statistically significant differences (FDR corrected p<0.05) are in bold. Up-regulated values are in blue and down-regulated values are in yellow.

In a previous transcriptome analysis of potato HR responses to PVY infection [24], we checked the expression profile of identified potato *MKK*s [35]. According to the gene expression profiles obtained for the potato cv. Rywal, three out of five *MKK*s showed differential expression for the HR response to PVY: St*MKK3*, St*MKK6* and St*MKK4/5* (Figure 2). St*MKK6* showed the strongest response of all, thus its expression data were also confirmed by quantitative real-time PCR (qPCR) (Table S2 in File S1). St*MKK6* was strongly up-regulated before lesion formation (3 dpi). In contrast, at the same time, St*MKK3* was down-regulated. St*MKK4/5* showed two-fold induction at the later time (6 dpi) following inoculation. For the SA-deficient plants (NahG-Rywal), the regulation of these *MKK*s at the gene level was either attenuated (for St*MKK3* and St*MKK4/5*) or delayed (for St*MKK6*). Only St*MKK7/9* was induced earlier and stronger in the NahG-Rywal plants, compared to the non-transgenic plants.

Based on these data we focused on further investigations on the At*MKK6* orthologue in potato, St*MKK6.*

### St*MKK6* orthologues across the plant kingdom

**Figure 3:**
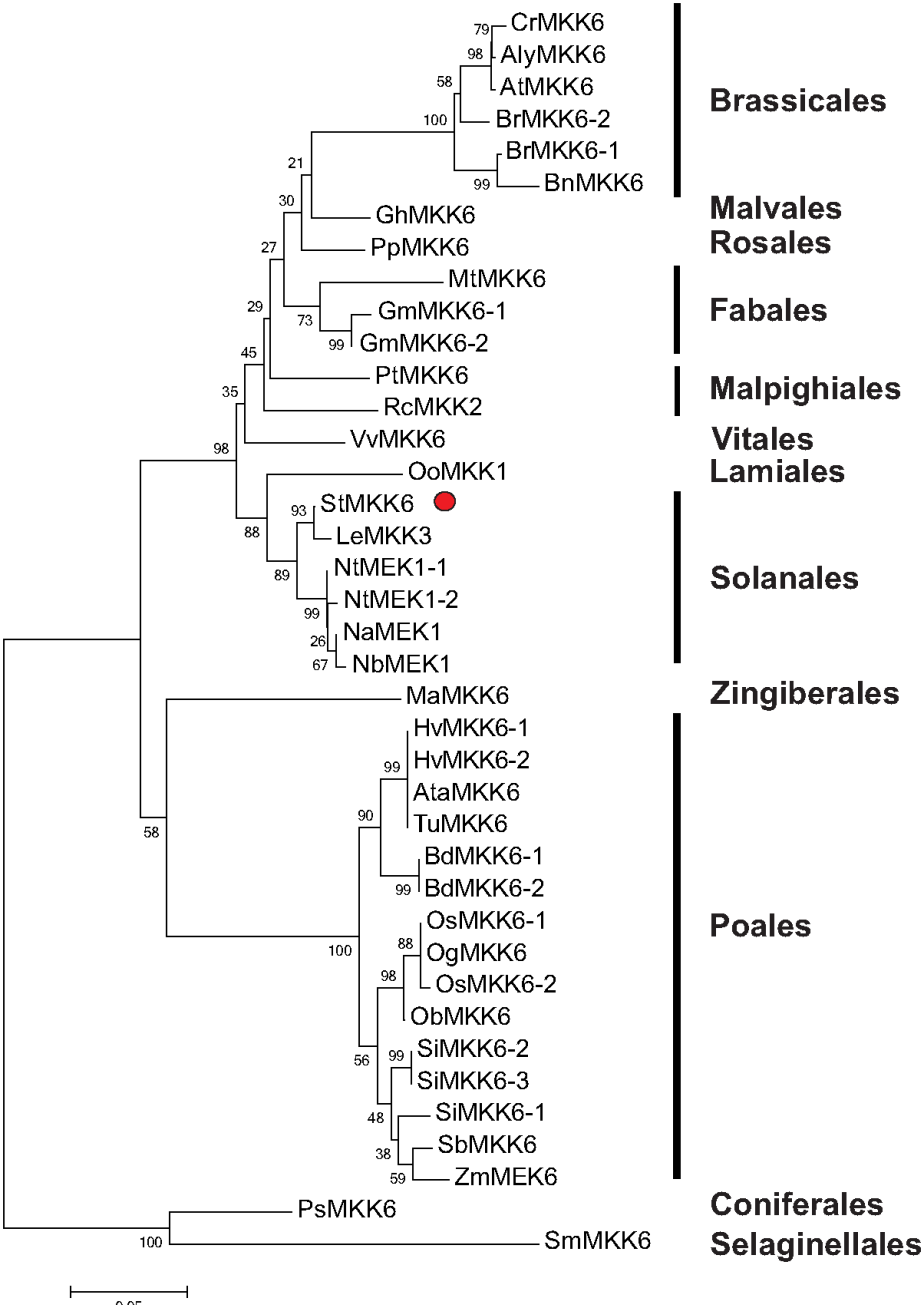
Phylogenetic tree of potato *MKK6* and its orthologues in different plant species, divided into classes. The species of origin for each *MKK* is marked by species acronym before the protein name: Cr, *Capsella rubella*; Aly, *Arabidopsis lyrata*; At, *Arabidopsis thaliana*; Br, *Brassica rapa*; Bn, *Brassica napus*; Gh, *Gossypium hirsutum*; Pp, *Prunus persica;* Mt, *Medicago trunculata*; Gm, *Glycine max*; Pt, *Populus trichocarpa*; Rc, *Ricinus communis*; Vv, *Vitis vinifera*; Oo, *Origanum onites*; St, *Solanum tuberosum*; Le, *Solanum lycopersicum* (*Lycopersicum esculetum*); Nt, *Nicotiana tabacum*; Na, *Nicotiana attenuata*; Nb, *Nicotiana benthamiana*; Ma, *Musa acuminata*; Hv, *Hordeum vulgare*, Ata, *Aegilops tauschii*; Tu, *Triticum urartu*; Bd, *Brachypodium distachyon*; Os, *Oryza sativa*, Og, *Oryza glaberrima*; Ob, *Oryza brachyantha*; Si, *Setaria italica*; Sb, *Sorghum bicolor*; Zm, *Zea mays,* Ps, *Picea sitchensis*; Sm, *Selaginella moellendorffii.* Potato *MKK6* is marked with red dot. The numbers on the nodes are percentages from a bootstrap analysis of 1000 replicates. The scale bar indicates the branch length that corresponds to 0.06 substitutions per site.

To study the sequence diversity and evolution of the *MKK6* gene, we performed a phylogenetic analysis within the plant kingdom. We identified 39 plant *MKK6* orthologues among higher plants. As well as for different families of Angiosperms, one *MKK6* gene was also identified in Gymnosperms, and one in mosses (Figure 3 and File S3). *MKK6* is mostly a single-copy gene, as these 39 *MKK6* orthologues belong to 31 plant species (Figure 3).

Comparison of the *MKK6* sequence from potato cv. Rywal and the published potato genome sequence of *S. tuberosum* Group Phureja, Clone DM showed that the coding domain sequences are different for three nucleotides at different sites, which results in only one amino-acid difference; this is not part of the protein kinase domain.

### Regulation of St*MKK6* gene expression in development and stress

To better understand the function of the *MKK6*s, we examined the gene expression data that is available for *A. thaliana MKK6* in Genevestigator. At*MKK6* is up-regulated in developmental processes (callus formation, germination) and after treatments with translation inhibitor cyclohexamide and hormones auxins. At*MKK6* is also up-regulated after flagellin 22 treatment, and under abiotic stress (cold, salt, acidic pH, hypoxia). At*MKK6* is down-regulated after treatments with abscisic acid and combinations of abscisic acid with jasmonate or SA.

**Figure 4:**
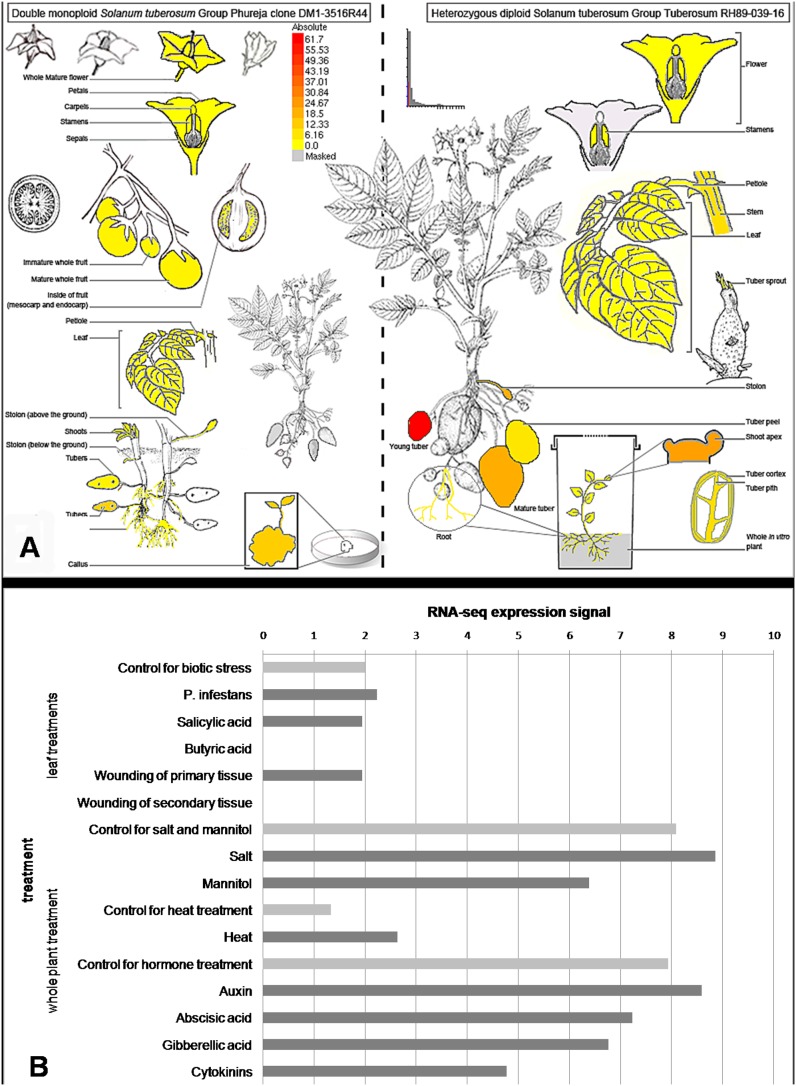
St*MKK6* gene expression in development and stress [40]. Data are obtained from the potato eFP browser [39]. **A.** Tissue and developmental gene expression pattern of St*MKK6* in double monoploid *S. tuberosum* Group Phureja (left) and in heterozygous diploid *S. tuberosum* Group Tuberosum (right). **B.** Changes in St*MKK6* gene expression under biotic stress conditions (treatments of leaves with *P. infestans*, SA analogues acibenzolar-S-methyl and fungal elicitor DL-β-amino-n-butyric acid and wounding) and abiotic stress conditions (treatments of whole plants with salt, heat and hormones cytokinins, 6 benzylaminopurine; gibberellins, GA3; abscisic acid; and auxin, IAA) and control treatments.

The expression profiles of St*MKK6* obtained from the Potato eFP Browser database showed that in the potato Group Phureja Clone DM, St*MKK6* tissue expression was high in the tubers and callus, and very low (below detection levels) in the flowers. In the Group Tuberosum Clone RH under non-stress conditions, St*MKK6* gene expression was strong also in the shoot apex (Figure 4A; [40]). Among all of the stress-related conditions included, compared to the controls, St*MKK6* was down-regulated after wounding of the secondary leaves and after treatment with the fungal elicitor DL-β-amino-n-butyric acid (Figure 4B).

### Analysis of the St*MKK6* native promoter

**Figure 5:**
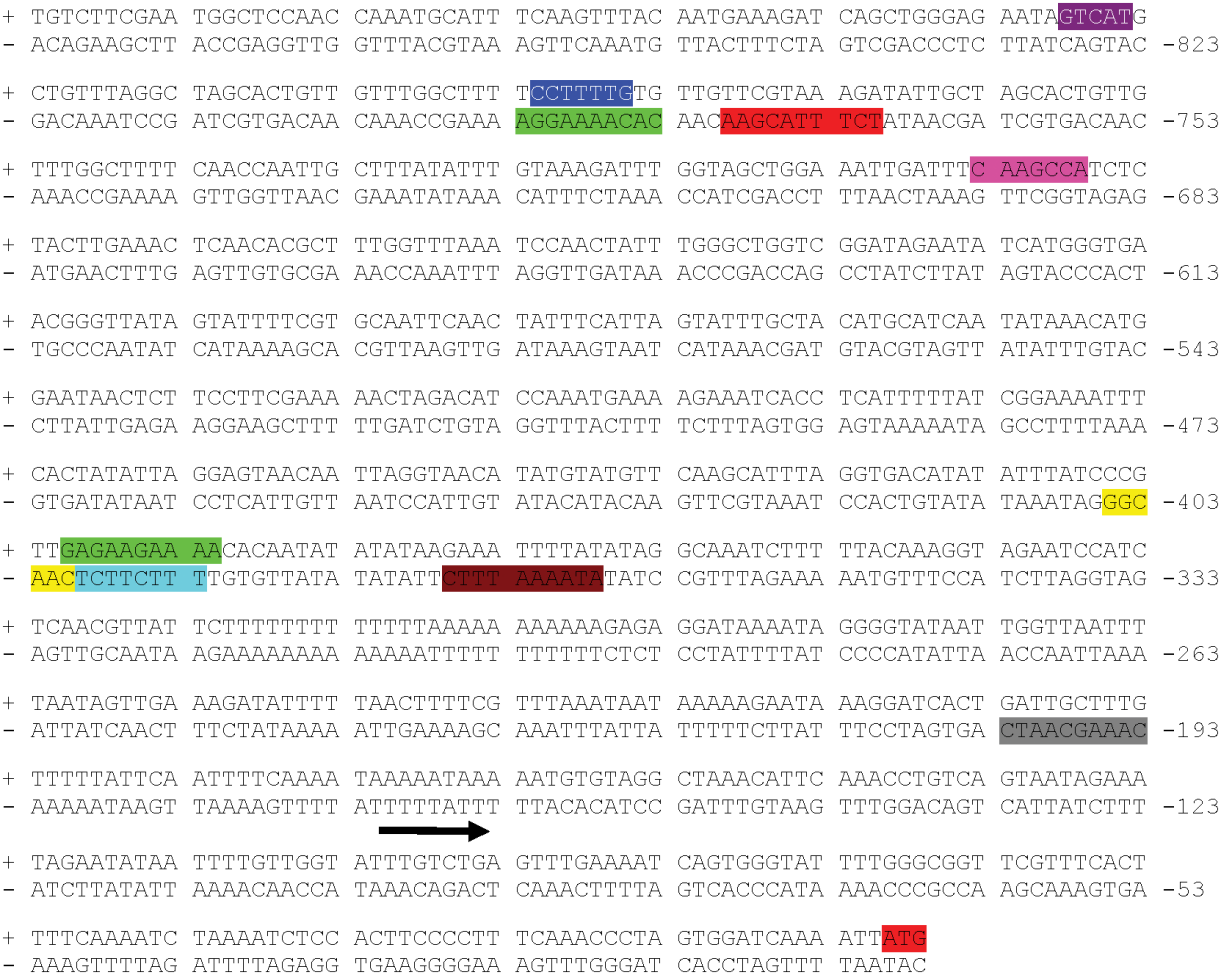
Predicted regulatory domains of St*MKK6* native promoter from *S. tuberosum* cv. Rywal, 790-bp-long. The crucial domains that regulate stress responses and development are CCAAT-box (MYBHv1 binding site; yellow), GCN4 motif (endosperm expression; pink), HSE (heat stress; brown), P-box (gibberellins; blue), Skn-1 motif (endosperm expression; purple), TCA element (salicylic acid; green), WUN motif (wounding; red) and circadian motif (control of circadian clock; grey). The arrow indicates the predicted beginning of the 5’ UTR region and the red shaded ATG indicates the start of St*MKK6* coding region. The complete list of St*MKK6* promoter domains from cv. Santé and cv. Rywal with their position, strand and sequence are specified in Tables S3 and S4 in File S1.

In the Potato Genome Sequencing Consortium (PGSC) Potato Genome Browser, St*MKK6* is assigned to PGSC gene ID PGSC0003DMG403005720, and it is localised to chromosome 3. Based on this information, we also cloned the St*MKK6* promoter sequence from two cultivars of *S. tuberosum* Group Tuberosum, both of which are resistant to PVY infection: cv. Rywal (GenBank accession number KF837128) and cv. Santé (GenBank accession number KF837129). The 790 bp-long promoter isolated from cv. Rywal (Table S3 in File S1) is identical to the promoter of *S. tuberosum* Group Phureja, but different from that of cv. Santé (Table S4 in File S1), due to a 27-bp-long region that is localised 230 bp upstream of St*MKK6*. In general, the most common domains were the TATA-box, CAAT box and domains related to light responsiveness. Based on the development and stress-related domains found, St*MKK6* appears to be involved in development of the endosperm and in responses to heat stress and wounding. St*MKK6* is also predicted to be involved in gibberellic acid and salicylic acid signalling, and is under regulation by the circadian clock (Figure 5).

### Regulation of St*MKK6* by salicylic acid

Promoter analysis of St*MKK6* has demonstrated potential involvement of St*MKK6* in SA signalling. Therefore, we examined expression of St*MKK6* under the SA treatment. The expression of St*MKK6* was measured by qPCR 24 h after treatment with SA in Rywal and NahG-Rywal plants.

**Figure 6:**
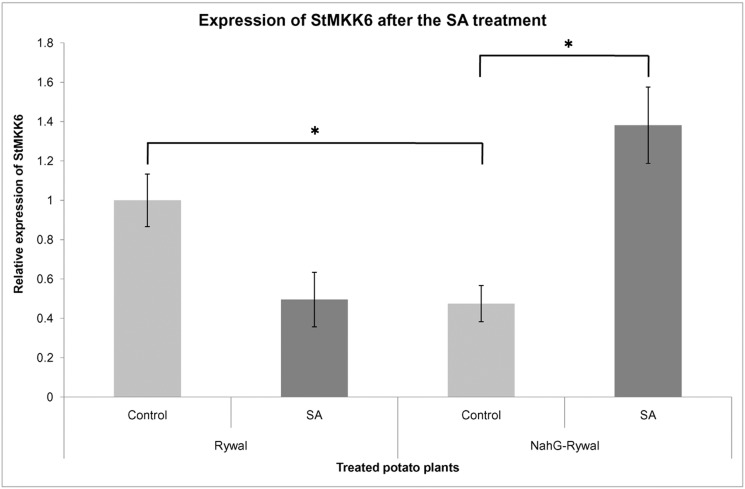
Expression of St*MKK6* in control and SA-treated Rywal and NahG-Rywal potato plants. Expression of the endogenous St*MKK6* in NahG-Rywal is at least two-fold lower than in Rywal. After the SA-treatment the expression of St*MKK6* in NahG-Rywal increased for at least three times, comparing to the non-treated control. Difference in the endogenous St*MKK6* expression between Rywal and NahG-Rywal as well as difference in the St*MKK6* expression between SA-treated and non-treated NahG-Rywal plants are statistically significant (p<0.05).

The treatment showed St*MKK6* to be sensitive to the present active form of SA since significant changes of St*MKK6* expression were observed in the SA-deficient genotype NahG-Rywal (Figure 6). There was a substantial, two-fold rise, of the St*MKK6* RNA, compared to the non-treated plants. In the Rywal plants, that received the same SA treatment, the change in the St*MKK6* expression was not significant (Table S5 in File S1).

We also observed significant difference in the RNA level of the endogenous St*MKK6* in the non-treated Rywal, compared to the NahG-Rywal (Figure 6). In Rywal plants there was at least twice as much of expressed St*MKK6* comparing to the level in the NahG-Rywal (Table S6 in File S1).

### Localisation of the StMKK6 protein in healthy and PVY-infected epidermal leaf cells

**Figure 7:**
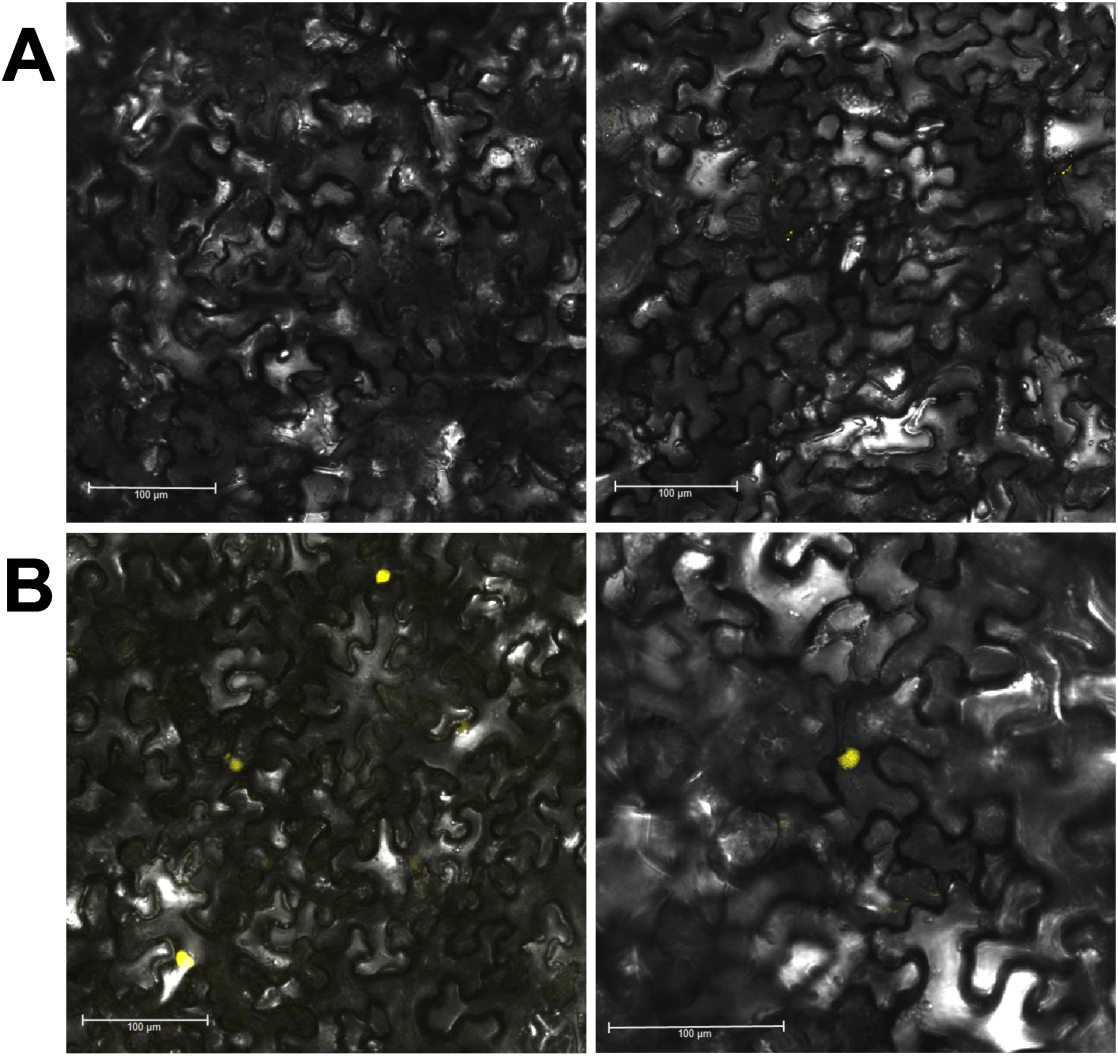
Epidermal cells of *N. benthamiana* expressing translational fusion of StMKK6 with YFP under native promoter. Leaves were agroinfiltrated when the virus has spread uniformly through the inoculated leaves (8 days after inoculation) and observed after 72 h in two independent experiments. Examples from two plants (left and right panels) are shown. Control of transformation (fluorescent marker without StMKK6 fusion) is in the Figure S1A. **A.** Localisation of StMKK6 in mock-inoculated leaves. No fluorescence was observed. **B.** Localisation of StMKK6 in PVY-inoculated leaves, where the protein accumulates predominantly in nucleus. Additional images of StMKK6 localisation under native promoter are in Figures S1D and S1E.

To learn more about the function of the StMKK6 protein in defence responses against the virus PVY, we analysed StMKK6 localisation in healthy and infected *N. benthamiana* plants. The infected leaves of *N. benthamiana* developed no necrotic lesions due to infection with PVY or overexpression of St*MKK6*. *In-silico* analysis predicted the localisation of StMKK6 as in the nucleus and cytoplasm. The same results were obtained also for AtMKK6 (Table S7 in File S1). An *in-vivo* study of the subcellular localisation of StMKK6 was performed under the native promoter and in C-terminal translational fusion with YFP in *N. benthamiana* leaves. In the mock-inoculated leaves, no fluorescence of the StMKK6-YFP fusion protein was observed (Figure 7A), while in PVY-infected leaves, there was strong StMKK6-YFP fluorescence in the nucleus and very weak StMKK6-YFP fluorescence in the cytoplasm (Figure 7B).

The fluorescent protein itself did not affect the localisation of StMKK6, as fusions with different fluorescent proteins were observed in the same intracellular compartments. The overexpression of StMKK6, driven by the CaMV 35S promoter, resulted in localisation of the protein in the cytoplasm and nucleus in mock-inoculated as well as in PVY-infected leaves (Figures S1B and S1C).

### Search for St*MKK6* co-regulated genes and potential targets in the MAPK cascade

To further improve our knowledge of St*MKK6* function, we searched for the other components of the MAPK module that is involved in the HR response in potato, the genes that are co-expressed with St*MKK6*, and the possible MAPK targets.

We found 22 co-expressed genes (File S4), for which we analysed the promoter regions. The most common biotic stress-related domains in their promoters were TC-rich repeat motifs (an element involved in defence and stress responsiveness; 13 promoters with the region), TGACG and CGTCA motifs (regulatory elements involved in methyl jasmonate responsiveness; 9 promoters with the region), Box-W1 (fungal elicitor responsive element; 8 promoters with the region), and TCA elements (SA responsiveness; 8 promoters with the region). The only region that was common to all 22 genes was the Skn-1 motif, which is related to endosperm expression.

In an *A. thaliana* interactome study [34], AtMKK6 was shown to interact with: AtMAPK4 (AT4G01370), AtMAPK6 (AT2G43790), AtMAPK11 (AT1G01560), AtMAPK12 (AT2G46070), AtMAPK13 (AT1G07880), and the calmodulin-like protein TCH3 (AT2G41100).

**Figure 8:**
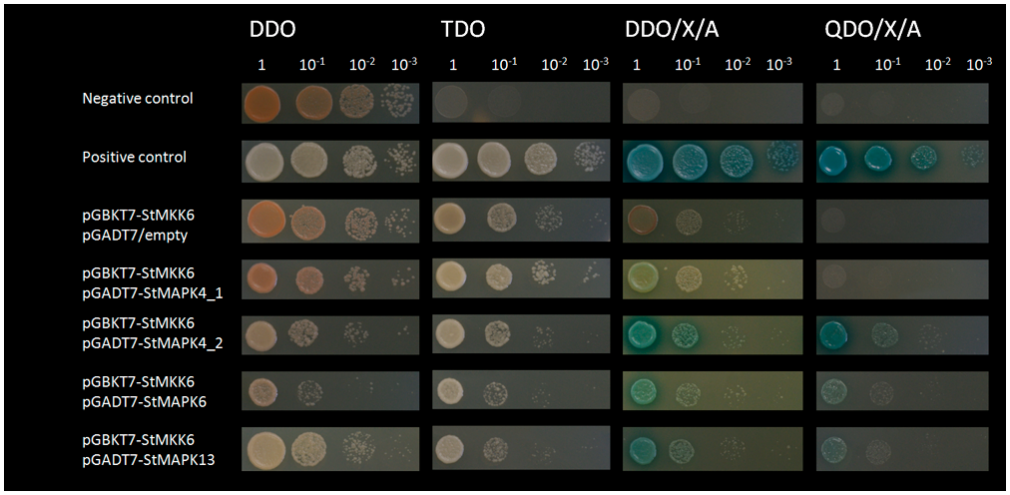
Yeast two-hybrid assays screening StMKK6 interaction partners. StMKK6 protein was fused with Gal DNA-binding domain as bait and StMAPK4_1, StMAPK4_2, StMAPK6 and StMAPK13 were used as prey fused with Gal DNA-activation domain. Interaction pairs P53/SV40 large T-antigen and Lam/SV40 large T-antigen were used as positive and negative controls respectively. StMKK6/empty vector pair was used as a control to discard auto-activation of bait protein. Serial dilutions of interaction pairs were plated on DDO media for co-transformation selection. Interactions of bait and prey proteins were examined by assessing growth on several selective media with different levels of restrictiveness i.e. SD/-Leu/-Trp/-His (TDO), SD/-Leu/-Trp/x-a-Gal/Aba (DDO/X/A) and QDO/X/A. Only co-transformed colonies growing on the most restrictive media (QDO/X/A) were considered as positive interaction transformants.

We first identified the orthologues of all of the potential interacting MAPKs in potato. AtMAPK4 together with AtMAPK11 and AtMAPK12 represent one orthologue cluster with StMAPK4_1 (PGSC003DMP400037535) and StMAPK4_2 (PGCS003DMP400000144). We also identified specific orthologues of AtMAPK6 in Sotub08g010260.1.1, and for AtMAPK13 in PGSC003DMP400044003 (Figure S2). We further cloned all of the potential MAPK interactors of StMKK6 from potato cv. Rywal. Comparisons of the four MAPKs cloned from potato cv. Rywal and those published in the potato genome sequence of *S. tuberosum* Group Phureja, Clone DM showed Rywal MAPK4_1 to be one-amino-acid different from PGSC0003DMP400037535, while Rywal MAPK 4_2 and MAPK13 are identical to their Phureja orthologues. Rywal MAPK6 had no corresponding proteins in Group Phureja, Clone DM. Yeast two-hybrid assays confirmed the interactions of StMKK6 with three MAPKs: StMAPK4_2, StMAPK6 and StMAPK13 (Figure 8). StMAPK4_1 could not be confirmed as a target of StMKK6 in potato cv. Rywal.

**Figure 9:**
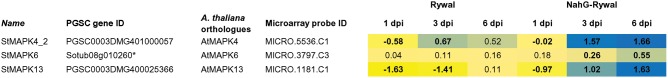
Gene expression pattern of confirmed StMKK6 targets in the HR response against PVY. The experimental setup is as in Figure 2. To each probe an *A. thaliana* orthologue, potato orthologue and a PGSC gene ID were assigned. Log2 of gene expression differences between PVY-infected and mock-inoculated plants are indicated for each time point (1, 3 and 6 dpi). Statistically significant differences (FDR corrected p-value <0.05) are given in bold. The star (*) for StMAPK6 PGSC gene ID indicates that the gene was not predicted in PGSC gene model. Up-regulated values are in blue and down-regulated values are in yellow.

St*MAPK4_2* and St*MAPK13* show differential gene expression patterns after PVY inoculation (Figure 9), and have different expression patterns than St*MKK6* (Figure 2).

## Discussion

To shed further light on the hypersensitive resistance signalling responses during PVY infection of potato, we analysed the involvement of potato MKKs in this process. The results revealed that the St*MKK6* is regulated by the SA signalling pathway in the HR to PVY infection. The virus induces the expression of St*MKK6* by the protein accumulating preferentially in nucleus.

### Potato has five *MKK*s

To date, MKK genes have been analysed in several plants: in *A. thaliana*, 10 have been identified [4], in rice, 8 [48], in *Brachypodium*, 12 [49], in poplar, 10 [50], in apple, 9 [51] and in canola, 7 [52]. In the *Solanaceae* family, the MKK family is smaller than in other plant species. Our analysis showed that the potato and tomato genomes have five MKK genes, and that the *N. benthamiana* genome has eight. Five *MKK*s were identified also in *N. attenuata*, while in *N. tabacum*, an additional Nb*MKK1* orthologue was not found among the currently existing sequences (Figure 1, [53]). The phylogenetic tree of the different MKK genes shows that only *N. benthamiana* has three possible pairs of paralogues, with none found in potato or tomato. Higher numbers of *MKK* family members in other plant species might be the result of gene duplications in the *MKK* ancestors that occurred after divergence into different families. Consequently, it was not possible to assign a single *A. thaliana* orthologue to three out of five potato *MKK*s. This issue of assigning orthologues was previously discussed by Dóczi et al. 2012 [54], and was attributed to the high expansion and functional diversification of the MAPKs, and to the rapid evolution of MAPK signalling.

It has been speculated that all 10 AtMKKs are fully functional, although there have been doubts expressed for AtMKK8 and AtMKK10. Although AtMKK8 has all of the typical MKK motifs, there is no evidence of its expression, while AtMKK10 lacks a correctly constructed target site in the activation loop [7]. Thus, in plants with lower numbers of MKK genes, the plasticity of the MAPK signalling module is not necessarily also proportionally lower.

### StMKK6 preferentially accumulates in the nucleus only after PVY infection

Transcriptomic analysis indicated that the St*MKK6* gene is involved in the HR response against PVY, as the most intensively regulated of the *MKK*s (Figure 2). Thus, we focused on different aspects of St*MKK6* function in this process. Here, meta-analysis of the publicly available datasets (Figure 4) and our *in-planta* localisation study (Figure 7) show that St*MKK6* expression is very low in non-stressed potato leaves. Interestingly, the infection with PVY induces the expression of St*MKK6*, with the StMKK6 protein concentrated predominantly in nucleus.

The information on the intracellular localisation of StMKK6 and its orthologues is currently really limited. The only report on experimental results of MKKs intracellular localisation showed that AtMKK6 accumulated in the equatorial plane of the phragmoplast in dividing root epidermal cells, while all of the other AtMKKs (AtMKK1-5) did not [10]. In *N. benthamiana,* localisation of MKK1 (an orthologue of AtMKK7, -8 and -9) was studied for the HR to *Phytophtora infestans* infection [55], [56]. *N. benthamiana* MKK1 preferentially accumulated in the nucleus also under non-stress conditions. Liang et al. 2013 [52], studied the localisation of MKK2, -3 and -4 in *Brassica napus* epidermal leaf cells, and detected the accumulation of these MKKs both in the cytoplasm and the nucleus. On the other hand, other reports on the localisation of the MKK target MAPKs show preferential accumulation of those proteins in the cytoplasm [52], [55]. One however has to note that all above mentioned experiments were studying localisation of MKKs when expressed under strong (e.g. CaMV 35S) promoter, while we have studied localisation under its native promoter (Figure 7). Also in our hands expression of protein under 35S promoter caused ubiquitous cellular localisation (e.g. cytoplasm and nucleus, see Figures S1B and S1C) presumably due to excessive amounts of protein produced, which is known to generate localisation artefacts [57]. Anyhow, our results support the hypothesis that activation of MKK6 targets occurs predominately in the nucleus as indicated also by *in silico* localisation prediction of identified target MAPKs and that its activitiy is regulated on the level of MKK6 transcription.

Several studies have focused on the gene expression profiles of St*MKK6* orthologues in other species. *A. thaliana* At*MKK6* and maize *MKK6* (Zm*MEK1*) are involved in the regulation of cytokinesis [10], [58], [59] and are required for lateral root formation [60]. Zm*MEK1* is also induced by polyethylene glycol, abscisic acid and SA, and it is down-regulated by NaCl [61]. In rice, Os*MEK1* (referred to as Os*MKK6* in Hamel et al. 2006 [7]) was reported to be involved in signalling of moderately low temperature stress [62], [63]. The present investigation into St*MKK6* expression in different tissues of the *S. tuberosum* group Phureja shows that St*MKK6* is highly expressed in tissues with intensive cell proliferation, similar to what has been reported for its orthologues in *A. thaliana*, tobacco and maize [10], [58], [59], [64].

After the SA treatment of the NahG-Rywal, the level of St*MKK6* transcripts three-fold increased, while in non-transgenic Rywal we detected trend of decrease in transcription of StMKK6 gene (Figure 6). Higher basal SA levels in cv. Rywal could explain different regulation of St*MKK6* after exogenous treatment. In fact, our results showed that basal levels of St*MKK6* transcripts in Rywal are at least two-fold higher than in tha SA-deficient NahG-Rywal which indicates that SA is necessary for the maintenance of basal levels of the St*MKK6*. This effect was previously reported for other SA-dependent defence-related genes in cv. NahG-Rywal [24] and cv. NahG-Desirée [23]. The network of SA signalling is complex [23], [65]–[67] and expected to show some nonlinear behaviour. Consequently the response we see might be both time as well as concentration dependent and we will not be able to fully predict the outcome of SA signalling before its appropriate dynamic model exisits. In line with hypothesis that St*MKK6* is regulated by SA, the analysis of the St*MKK6* promoter revealed motifs that are responsive to SA (Figure 5). To the best of our knowledge, there has been only one study linking St*MKK6* expression and SA signalling for any of the St*MKK6* orthologues (Zm*MEK1*) [61]. In *B. napus*, *MKK1, MKK2, MKK4* and *MKK9* were induced by SA, while only *MKK3* was not [52], which indicates that the majority of MKKs are targets of the SA signalling network.

The microarray results show that only *MKK3* (group B *MKK*s) responded in the opposite direction; i.e., *MKK3* was repressed after viral infection, contrary to all of the other potato *MKK*s, which were induced. Similarly, it has been shown also for *A. thaliana* and tobacco, that the MKK3 acts as a negative regulator in the JA signalling pathway and response to herbivores [11], [16], [68].

### MAPK signalling network in hypersensitive resistance response to PVY

We identified here three downstream targets of StMKK6 in potato, StMAPK4_2 (orthologue of AtMAPK4, -11 and -12), StMAPK6 (orthologue of AtMAPK6 and tobacco SIPK) and StMAPK13 (orthologue of AtMAPK13 and tobacco NTF6/NRK1). All of these interactions had already been confirmed in *A. thaliana* [34], [69], [70]. In rice, OsMEK1 (or OsMKK6) interacts with OsMAPK1, -3, -5 and -6 (orthologues of AtMAPK6, -3 and -11/4, respectively [48], [63]). To date, only one MAPK interactor has been identified for the MKK6 orthologues in *Solanaceae*: in *N. tabacum*, NQK1/NtMEK1 was reported to interact with NTF6/NRK1 (orthologue of AtMAPK13 [64]). According to this, we can conclude that the MKK6 signalling module is evolutionarily relatively stable, as most of the interactions appear to be conserved between unrelated species. There are, however, some specificities. For example, out of two potato MAPK4/11/12 paralogues StMKK6 interacts with only one of them, while in *A. thaliana* it interacts with all three, and in rice, with two.

Among the StMKK6 downstream targets, the present study shows that only St*MAPK13* expression (Figure 9) is significantly regulated in the HR response to PVY infection, although while St*MKK6* is strongly up-regulated, St*MAPK13* is strongly down-regulated. Interestingly St*MAPK4_2* and St*MAPK13* are significantly induced in SA deficient plants at the same time points as St*MKK6*. All of the target MAPKs are, however, expressed at (substantially) higher levels in both the mock-infected and PVY-infected leaves, compared to St*MKK6* (Figure S3). Therefore, we can hypothesise that the crucial regulation of St*MAPK4_2*, *6* and *13* is not at the gene expression level, but at a later step; e.g., phosphorylation by newly synthesised StMKK6, or any other post-translational process (e.g. protein translocation or degradation).

As well as St*MKK6*, we showed that St*MKK4/5* (also named as St*MEK1* in some studies) is also up-regulated in the HR response of potato to PVY, albeit to a lower extent (slightly below the strict significance cut-off), and showed a delay comparable to St*MKK6* in the NahG plants (Figure 2). In *N. tabacum*, both Nt*MEK2* (orthologue of St*MKK4/5*) and *NQK1*/Nt*MEK1* (orthologue of St*MKK6*) are required for N-mediated resistance against tobacco mosaic virus [71]–[73]. Similarly, the silencing of At*MEK1* and At*MEK2* allowed for higher amplification of *Cucumber mosaic virus* in *A. thaliana* [74]. This indicates the close connectivity and interdependency between the St*MEK1* and St*MEK2* signalling modules in plant defence.

Since an effective HR takes place in cv. Rywal in response to PVY, it is possible that some of the regulated potato MKKs are part of this response. A comprehensive analysis of the different *MKK* functions was performed in Pto-mediated resistance in tomato by Ekengren et al., 2003 [75], and Pedley and Martin, 2004 [76]. Ekengren et al. [75] showed that silencing of either *MEK1* or *MEK2* breaks the resistance against *Pseudomonas syringae*. However, Pedley and Martin [76] showed that Le*MKK2* (orthologue of At*MKK4/5*) and Le*MKK4* (orthologue of At*MKKK7/8/9*) caused programmed cell death when overexpressed, while Le*MKK1* (orthologue of At*MKK1/2*) and Le*MKK3* (orthologue of *MKK6*) did not. In *A. thaliana*, two studies have shown that as well as the MKK4/MKK5 (MEK2) module, another branch of the group A MAPKKs, AtMKK1 and AtMKK2, function together as the second MAPK module involved in the HR response [68], [77]. In *N. benthamiana MKK1* (orthologue of At*MKK7/8/9*) is a potent inducer of HR-like cell death in response to *P. infestans* [11], [55], [78] and the same was shown also for At*MKK7* and *MKK9* in *A. thaliana* [79]. In *A. thaliana*, *MKK5* was shown to have a role in the cascade that triggers the HR response [80]. In the present study, there was strong repression of potato *MKK3*, while this dysregulation was diminished in the NahG-Rywal plants (Figure 2).

The interconnectivity between the NbMEK1 (orthologue of AtMKK6) and NbMEK2 (orthologue of AtMKK4/5) kinase signalling modules was studied mechanistically by del Pozo et al., 2004 [81]. They showed that the silencing of Nb*MEK1* abolished the cell death caused by constitutively active Nb*MEK2*. In *N. attenuata*, *MEK1* (orthologue of St*MKK6*) and *SIPKK* (orthologue of At*MKK1/2*) are involved in the regulation of the accumulation of 12-oxo-phytodienoic acid and JA [53], which from another point, supports the role of SA in MKK6 signalling, due to the known antagonistic cross-talk between JA and SA in defence pathways [82].

## Conclusions

Our results show that StMKK6 is an important player in potato HR response against PVY infection, as shown on the gene-expression level and protein localisation. We identified potential StMKK6 downstream targets and have shown that albeit the signalling network seems to be evolutionary relatively stable the fine-tuned interdependency within the broader MAPK signalling network might be different in different plants, and when a plant is exposed to different pathogens.

## Supporting Information

Figure S1 **Localisation of StMKK6, under 35S promoter and native promoter, in epidermal cells of** ***N. benthamiana*** **. A.** Control of transformation. Epidermal cells, transformed with plasmids containing 35S::pH7CWG2-CFP (left) and 35S::pH7YWG2-YFP (right) fusion. The fluorescence of the CFP or YFP alone (without the fusion with StMKK6) is observed only in cytoplasm. **B.** Localisation of StMKK6 fused with CFP (upper panel) or YFP (lower panel) with expression under the CaMV 35S promoter in mock-inoculated epidermal cells. The protein is localised in cytoplasm and nucleus. **C.** Localisation of StMKK6 fused with CFP (upper panel) or YFP (lower panel) with expression under the CaMV 35S promoter in PVY-inoculated epidermal cells. The protein is localised in cytoplasm and nucleus. **D.** Localisation of StMKK6 fused with YFP with expression under native promoter in mock-inoculated epidermal cells, where no fluorescence is observed. **E.** Localisation of StMKK6 fused with YFP with expression under native promoter in PVY-inoculated epidermal cells, where the protein accumulates predominantly in nucleus.
(TIF)

Figure S2 **Phylogenetic tree of potato and** ***A. thaliana*** **MAPKs from group A and group B.** Potato (Sotub, PGSC and St) and *A. thaliana* (At) MAPKs from group A (MAPK3, 6 and 10) and group B (MAPK4, 5, 11, 12 and 13) as was already proposed by Ichimura et al. 2002 [4]. Besides the potato sequences from the PGSC Browser (Sotub and PGSC) the tree also includes four MAPKs from cv. Rywal: StMAPK4_1, StMAPK4_2, StMAPK6 and StMAPK13. The sequences from *Arabidopsis* are AtMAPK3 (AT3G45640.1), AtMAPK4 (AT4G01370.1), AtMAPK5 (AT4G11330.1), AtMAPK6 (AT2G43790.1), AtMAPK10 (AT3G59790.1), AtMAPK11 (AT1G01560.1), AtMAPK12 (AT2G46070.1) and AtMAPK13 (AT1G07880.2). The scale bar indicates the branch length that corresponds to 0.06 substitutions per site.
(TIF)

Figure S3 **Expression pattern of St** ***MKK6*** **and its confirmed targets St** ***MAPK4_2*** **, St** ***MAPK6*** **and St** ***MAPK13*** **in mock-inoculated plants.** Log2 of normalized signals for mock-inoculated plants 1 day post inoculation are shown. The expression is shown for four mock treated Rywal (R) and NahG-Rywal (nah) plants. In both sets of plants the expression of the *MKK6* interacting *MAPK*s is higher than of St*MKK6*. Calculated are differences in the expression between Rywal and NahG-Rywal plants.
(TIF)

File S1 **Table S1 in File S1. Oligonucleotides used for fragment isolation, qPCR and cloning.** In primers for cloning, the underlined parts of sequences are complementary to the destination plasmids. **Table S2 in File S1. Validation of St** ***MKK6*** **microarray (µarray) results by real-time PCR (qPCR).** To validate the microarray results for St*MKK6*, its expression was analysed by qPCR in the same RNA samples as for the microarray analysis. Log_2_ of ratio between virus- and mock-inoculated plants in cv. Rywal and NahG-Rywal 1, 3 and 6 days after PVY inoculation for St*MKK6* obtained by both methods are shown. Statistically significant values (p<0.05) are marked with bold. **Table S3 in File S1. Predicted regulatory domains of St** ***MKK6*** **native promoter from** ***S. tuberosum*** **, cv. Rywal.** The promoter sequence is 899 bp-long and analysed with PlantCare software [36]. **Table S4 in File S1. Predicted regulatory domains of St** ***MKK6*** **native promoter from** ***S. tuberosum*** **, cv. Santé.** The promoter sequence is 247 bp-long and analysed with PlantCare software [36]. **Table S5 in File S1. Expression values of** ***StMKK6*** **in control and SA-treated potatoes cv. Rywal and NahG-Rywal.** Two biological replicates per treatment were analysed. Relative expression values and fold-changes (compared to Rywal control) are shown in the table. Differences between control and SA-treated plants were statistically evaluated by t-test. **Table S6 in File S1.** **Comparison of** ***StMKK6*** **basal expression values in Rywal and NahG-Rywal plants.** Two biological replicates per treatment were analysed. Relative expression values and fold-changes (compared to Rywal control) are shown in the table. Differences between Rywal and NahG-Rywal plants were statistically evaluated by t-test. **Table S7 in File S1. Subcellular localisation prediction of StMKK6 and AtMKK6.** Amino acid sequence of AtMKK6 (GenBank accession number NM_125041.2) and StMKK6 (GenBank accession number KF837129.1) proteins were used as query in PredictProtein service. The localisation for each was predicted by three different prediction algorithms: PROSITE, LOCkey and LOCtree. For each subcellular prediction a confidence to the prediction is given.
(DOC)

File S2 **Gene expression of potato** ***MKK*** **family in different tissues and after several stress treatments.** For each *MKK*, the RNA expression signals (FPKM values), based on the RNA sequencing of double monoploid *Solanum tuberosum* Group Phureja clone DM1-3 (DM) [40] and Genome sequence and analysis of the tuber crop potato [30] were collected in the Potato eFP browser [39]. **A.** Tissue expression of MKK gene family in Clone DM and Clone RH. In bold are the expression values that are more than 2-times different from the average expression in all the organs. **B.** Expression of *MKK* gene family after several stress treatments. In bold are the expression values that are more than 2-times different from the controls.
(XLSX)

File S3 **St** ***MKK6*** **orthologs with corresponding UniProt IDs.** List of St*MKK6* orthologues across plant kingdom. Listed are the Uniprot IDs for the proteins, names of the proteins as in the phylogenetic tree (Figure 3) and the organism the gene was originally isolated from.
(XLS)

File S4 **Expression of genes co-regulated with St** ***MKK6*** **and their regulatory domains in promoter regions.** Cultivar Rywal (HR response, conferred by Ny-1 gene) and NahG-Rywal (impaired accumulation of SA) were analysed for whole transcriptome response 1, 3 and 6 dpi after PVY infection [24]. Log2 fold changes of PVY in infected vs. mock inoculated plants are indicated for each time point (1, 3 and 6 dpi). Statistically significant differences (FDR corrected p<0.05) are in bold. Higher expression values are blue and lower are yellow. The promoter sequences were obtained from the *Solanum_tuberosum*-PGSC_DM_v34_superscaffolds database. 1000 bp-long promoter sequences upstream of the gene were analysed by PlantCARE web service [36]. Biotic stress-related domains are coloured and the colours are explained in the legend on the right.
(XLS)
