# Supplementary figures and images for: Involvement of Potato (Solanum tuberosum L.) MKK6 in Response to Potato virus Y
Source: PLoS One. 2014 Aug 11;9(8):e104553. doi: 10.1371/journal.pone.0104553 (PMC4128675; doi:10.1371/journal.pone.0104553)

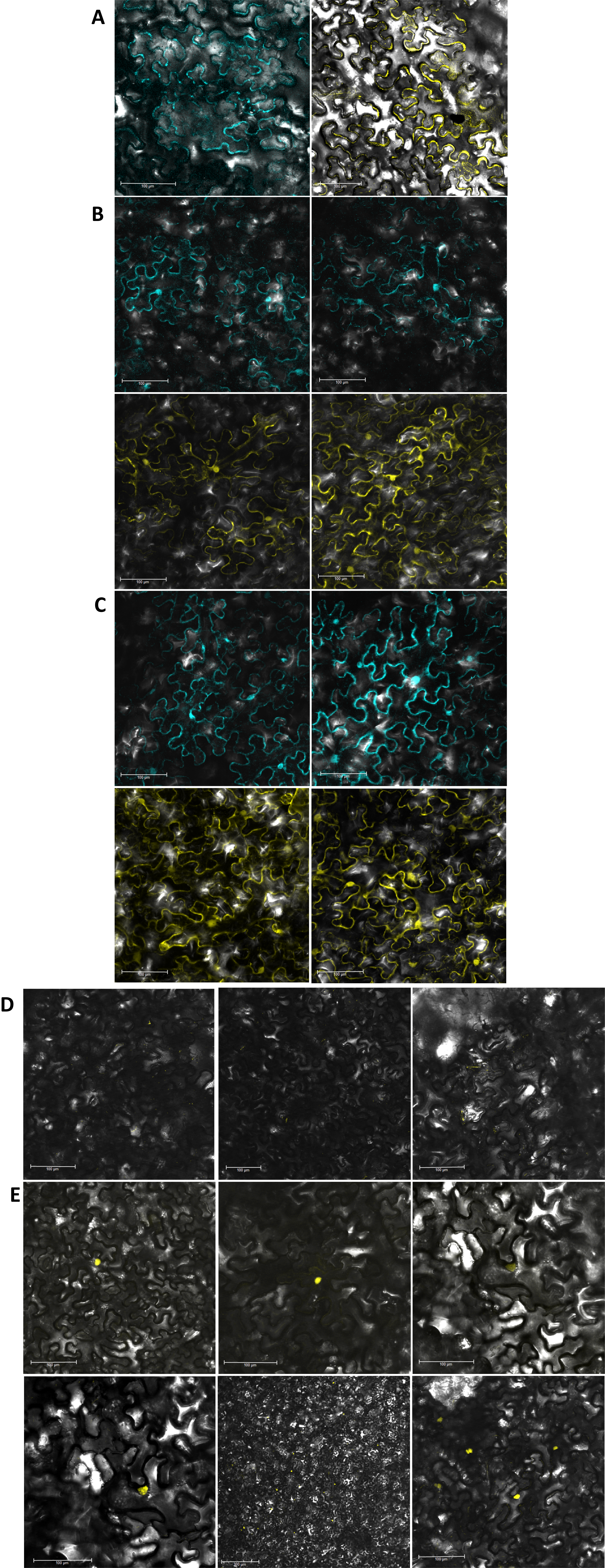

Supplement: Figure S1 — Localisation of StMKK6, under 35S promoter and native promoter, in epidermal cells of N. benthamiana . A. Control of transformation. Epidermal cells, transformed with plasmids containing 35S::pH7CWG2-CFP (left) and 35S::pH7YWG2-YFP (right) fusion. The fluorescence of the CFP or YFP alone (without the fusion with StMKK6) is observed only in cytoplasm. B. Localisation of StMKK6 fused with CFP (upper panel) or YFP (lower panel) with expression under the CaMV 35S promoter in mock-inoculated epidermal cells. The protein is localised in cytoplasm and nucleus. C. Localisation of StMKK6 fused with CFP (upper panel) or YFP (lower panel) with expression under the CaMV 35S promoter in PVY-inoculated epidermal cells. The protein is localised in cytoplasm and nucleus. D. Localisation of StMKK6 fused with YFP with expression under native promoter in mock-inoculated epidermal cells, where no fluorescence is observed. E. Localisation of StMKK6 fused with YFP with expression under native promoter in PVY-inoculated epidermal cells, where the protein accumulates predominantly in nucleus. (TIF) [file pone.0104553.s001.tif]

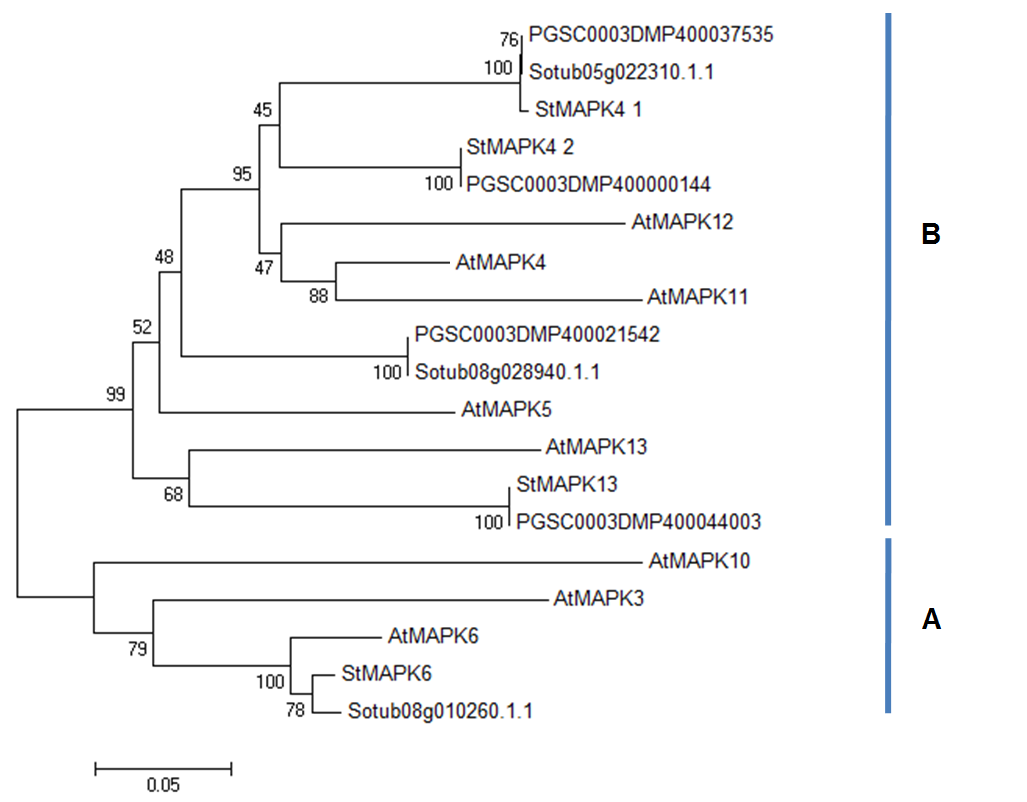

Supplement: Figure S2 — Phylogenetic tree of potato and A. thaliana MAPKs from group A and group B. Potato (Sotub, PGSC and St) and A. thaliana (At) MAPKs from group A (MAPK3, 6 and 10) and group B (MAPK4, 5, 11, 12 and 13) as was already proposed by Ichimura et al. 2002 [4]. Besides the potato sequences from the PGSC Browser (Sotub and PGSC) the tree also includes four MAPKs from cv. Rywal: StMAPK4_1, StMAPK4_2, StMAPK6 and StMAPK13. The sequences from Arabidopsis are AtMAPK3 (AT3G45640.1), AtMAPK4 (AT4G01370.1), AtMAPK5 (AT4G11330.1), AtMAPK6 (AT2G43790.1), AtMAPK10 (AT3G59790.1), AtMAPK11 (AT1G01560.1), AtMAPK12 (AT2G46070.1) and AtMAPK13 (AT1G07880.2). The scale bar indicates the branch length that corresponds to 0.06 substitutions per site. (TIF) [file pone.0104553.s002.tif]

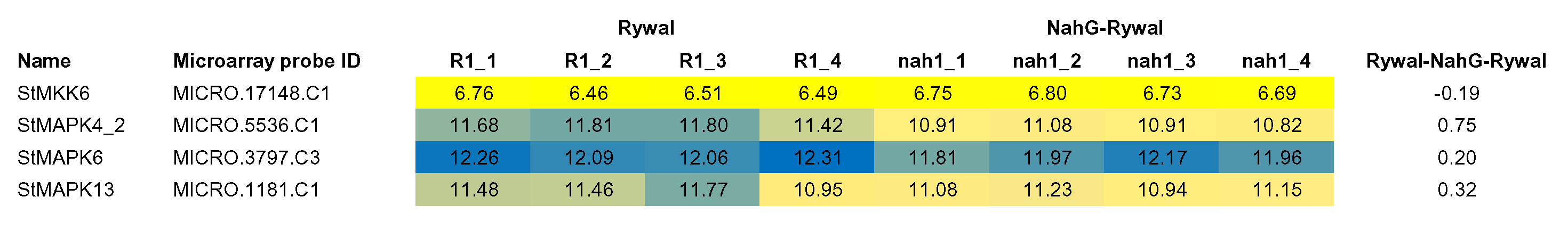

Supplement: Figure S3 — Expression pattern of St MKK6 and its confirmed targets St MAPK4_2 , St MAPK6 and St MAPK13 in mock-inoculated plants. Log2 of normalized signals for mock-inoculated plants 1 day post inoculation are shown. The expression is shown for four mock treated Rywal (R) and NahG-Rywal (nah) plants. In both sets of plants the expression of the MKK6 interacting MAPKs is higher than of StMKK6. Calculated are differences in the expression between Rywal and NahG-Rywal plants. (TIF) [file pone.0104553.s003.tif]
