# Supplementary material for: Involvement of Potato (Solanum tuberosum L.) MKK6 in Response to Potato virus Y
Source: PLoS One. 2014 Aug 11;9(8):e104553. doi: 10.1371/journal.pone.0104553 (PMC4128675; doi:10.1371/journal.pone.0104553)
Supplement: File S1 — Table S1 in File S1. Oligonucleotides used for fragment isolation, qPCR and cloning. In primers for cloning, the underlined parts of sequences are complementary to the destination plasmids. Table S2 in File S1. Validation of St MKK6 microarray (µarray) results by real-time PCR (qPCR). To validate the microarray results for StMKK6, its expression was analysed by qPCR in the same RNA samples as for the microarray analysis. Log2 of ratio between virus- and mock-inoculated plants in cv. Rywal and NahG-Rywal 1, 3 and 6 days after PVY inoculation for StMKK6 obtained by both methods are shown. Statistically significant values (p<0.05) are marked with bold. Table S3 in File S1. Predicted regulatory domains of St MKK6 native promoter from S. tuberosum , cv. Rywal. The promoter sequence is 899 bp-long and analysed with PlantCare software [36]. Table S4 in File S1. Predicted regulatory domains of St MKK6 native promoter from S. tuberosum , cv. Santé. The promoter sequence is 247 bp-long and analysed with PlantCare software [36]. Table S5 in File S1. Expression values of StMKK6 in control and SA-treated potatoes cv. Rywal and NahG-Rywal. Two biological replicates per treatment were analysed. Relative expression values and fold-changes (compared to Rywal control) are shown in the table. Differences between control and SA-treated plants were statistically evaluated by t-test. Table S6 in File S1. Comparison of StMKK6 basal expression values in Rywal and NahG-Rywal plants. Two biological replicates per treatment were analysed. Relative expression values and fold-changes (compared to Rywal control) are shown in the table. Differences between Rywal and NahG-Rywal plants were statistically evaluated by t-test. Table S7 in File S1. Subcellular localisation prediction of StMKK6 and AtMKK6. Amino acid sequence of AtMKK6 (GenBank accession number NM_125041.2) and StMKK6 (GenBank accession number KF837129.1) proteins were used as query in PredictProtein service. The localisation for each [file pone.0104553.s004.doc]

**File S1**

**Table S1. Oligonucleotides used for fragment isolation, qPCR and cloning. In primers for cloning, the underlined parts of sequences are complementary to the destination plasmids.**

| **Fragment isolation** | |
| --- | --- |
| StMKK6_F | 5-ATGAAGACGACGAAGCCATT-3 |
| StMKK6_R | 5-ATGAAGACGACGAAGCCATT-3 |
| MKK6_AP1 | 5-TGAAGATCTATCTCCTTAGTTTCTGA-3 |
| MKK6_AP2 | 5-TGAGTCTGAGTCCTTTCTGGTTCA-3 |
| MICRO.7088.C2 MAPK4_1 F | 5-TCCATTTTGGGGTTGATTTC-3 |
| MICRO.7088.C2 MAPK4_1 R | 5-TCTTTCAATGAGTTGGATCAGG-3 |
| MICRO.5536.C1 MAPK4_2 F | 5-ATGGAGGCAAGTTTAGGTGA-3 |
| MICRO.5536.C1 MAPK4_2 R | 5-TCTCTCAGTGAGTTGGATCTGG-3 |
| MICRO.3797.C3 MAPK6_F | 5-ATGGATGTTTCAGCTCCGCAAA-3 |
| MICRO.3797.C3 MAPK6_R | 5-TTCACATGCGCTGGTATTCA-3 |
| TA35254_4113_MAPK13_F | 5-AGAGCAGAAATGGATGCTGAA-3 |
| TA35254_4113_MAPK13_R | 5-TCACTTGGTTGTATCGGGATCAAAC-3 |
| **qPCR** | |
| MKK6_F qPCR | 5-AGCAGATCAATTTTCCCCAGAA-3 |
| MKK6_R qPCR | 5-GACTCAAAAGGTCCAAAGCTGAA-3 |
| MKK6_S qPCR | FAM/5-TCTGTTCGTTTGTTTCTGCTTGCATTCAAA-3/Zen Iowa BlackTM FQ |
| EF1_F | 5-GGAAGCTGCTGAGATGAACAAGA-3 |
| EF-1_R | 5-CTCACGTTCAGCCTTAAGTTTGTC-3 |
| EF1_S | FAM/5-TCATTCAAGTATGCCTGGGTGCT-3/TAMRA |
| COX_F | 5- CGTCGCATTCCAGATTATCCA-3 |
| COX_R | 5- CAACTACGGATATATAAGRRCCRRAACTG-3 |
| COX_S | FAM/5-TGCTTACGCTGGATGGAATGCCCT-3/TAMRA |
| **Cloning** | |
| MKK6_GW_F | 5-CACCATGAAGACGACGAAGCCATT-3 |
| MKK6_GW_R | 5-TCTTGGAAAATTTACTGGTGGTTCC-3 |
| pGADT7_ MAPK4_F | 5-ATGGCCATGGAGGCCAGTGAATTCCACATGTCTCTTGATTCAAGTTCAGCTGATCAT-3 |
| pGADT7_ MAPK4_R | 5-ATCTGCAGCTCGAGCTCGATGGATTCAATGAGTTGGATCAGGATTAAATTTTGC-3 |
| pGADT7_ MAPK4_2_F | 5-ATGGCCATGGAGGCCAGTGAATTCCACATGGAGGCAAGTTTAGGTGATCATGGT-3 |
| pGADT7_ MAPK4_2_R | 5-ATCTGCAGCTCGAGCTCGATGGATTCAGTGAGTTGGATCTGGATTAAAGTTCAC-3 |
| pGBKT7_MKK6_F | 5-ATCTCAGAGGAGGACCTGCATATGGCCATGAAGACGACGAAGCCATTGAAGCAA-3 |
| pGBKT7_MKK6_R | 5-AGAGGCCCCAAGGGGTTATGCTAGTTATGCTTATCTTGGAAAATTTACTGGTGGTTCCAG-3 |
| pGADT7_ MAPK6_F | 5-ATGGCCATGGAGGCCAGTGAATTCCACATGGATGTTTCAGCTCCGCAAATG-3 |
| pGADT7_ MAPK6_R | 5-ATCTGCAGCTCGAGCTCGATGGATTCACATGCGCTGGTATTCAGGATT-3 |
| pGADT7_ MAPK13_F | 5-ATGGCCATGGAGGCCAGTGAATTCCACATGGATGCTGAAAACATTGAAAATTCA-3 |
| pGADT7_ MAPK13_R | 5-ATCTGCAGCTCGAGCTCGATGGATTCACTTGGTTGTATCGGGATCAAACTT-3 |
| MAPKprom-YFP__F | 5-GCTAAGCTTGAGCTCTCCCATATGGTCACTATAGGGCACGCGTGGT-3 |
| MKK6prom-YFP__R | 5-TATCACTAGTGCGGCCGCCTGCAGGAATTTTGATCCACTAGGGTTTGA-3 |

**Table S2.** **Validation of St*MKK6* microarray (μarray) results by real-time PCR (qPCR).** To validate the microarray results for St*MKK6*, its expression was analysed by qPCR in the same RNA samples as for the microarray analysis. Log_2_ of ratio between virus and mock inoculated plants in cv. Rywal and NahG-Rywal 1, 3 and 6 days after PVY inoculation for St*MKK6* obtained by both methods are shown. Statistically significant values (p < 0.05) are marked with bold.

|  | **Rywal** | | | | | | **NahG-Rywal** | | | | | |
| --- | --- | --- | --- | --- | --- | --- | --- | --- | --- | --- | --- | --- |
|  | **1 dpi** | | **3 dpi** | | **6 dpi** | | **1 dpi** | | **3 dpi** | | **6 dpi** | |
|  | µarray | qPCR | µarray | qPCR | µarray | qPCR | µarray | qPCR | µarray | qPCR | µarray | qPCR |
| **St*MKK6*** | **1.40** | 0.37 | **1.74** | **1.29** | -0.17 | 0.14 | **0.90** | 0.21 | **0.77** | **1.18** | **0.65** | **2.54** |

**Table S3. Predicted regulatory domains of St*MKK6* native promoter from *S. tuberosum*, cv. Rywal. The promoter sequence is 899 bp-long and analyzed with PlantCare software [36].**

| **Site name** | **Organism** | **Position** | **Strand** | **Sequence** |
| --- | --- | --- | --- | --- |
| [5UTR Py-rich stretch](http://bioinformatics.psb.ugent.be/webtools/plantcare/cgi-bin/show_site_info.htpl?QWhere=ID_of_Site like 'LE~5UTR Py-rich stretch'&StartAt=0&NbRecs=10) **-** cis-acting element conferring high transcription levels | *Lycopersicon esculentum* | 497 | - | TTTCTTCTCT |
| ARE - cis-acting regulatory element essential for the anaerobic induction | *Zea mays* | 237 | + | TGGTTT |
| [Box I](http://bioinformatics.psb.ugent.be/webtools/plantcare/cgi-bin/show_site_info.htpl?QWhere=ID_of_Site like 'PS~Box I'&StartAt=0&NbRecs=10) - light responsive element | *Pisum sativum* | 718 | + | TTTCAAA |
|  | *Pisum sativum* | 846 | + | TTTCAAA |
|  | *Pisum sativum* | 807 | - | TTTCAAA |
|  | *Pisum sativum* | 874 | + | TTTCAAA |
| [CAAT-box](http://bioinformatics.psb.ugent.be/webtools/plantcare/cgi-bin/show_site_info.htpl?QWhere=ID_of_Site like 'BR~CAAT-box'&StartAt=0&NbRecs=10) - common cis-acting element in promoter and enhancer regions | *Brassica rapa* | 20 | + | CAAAT |
|  | *Hordeum vulgare* | 39 | + | CAAT |
|  | *Brassica rapa* | 122 | - | CAAAT |
|  | *Glycine max* | 129 | - | CAATT |
|  | *Hordeum vulgare* | 130 | - | CAAT |
|  | *Arabidopsis thaliana* | 159 | + | CCAAT |
|  | *Glycine max* | 160 | + | CAATT |
|  | *Glycine max* | 161 | - | CAATT |
|  | *Hordeum vulgare* | 162 | - | CAAT |
|  | *Brassica rapa* | 172 | - | CAAAT |
|  | *Brassica rapa* | 182 | - | CAAAT |
|  | *Glycine max* | 196 | - | CAATT |
|  | *Hordeum vulgare* | 197 | - | CAAT |
|  | *Brassica rapa* | 253 | - | CAAAT |
|  | *Glycine max* | 307 | + | CAATT |
|  | *Brassica rapa* | 328 | - | CAAAT |
|  | *Hordeum vulgare* | 343 | + | CAAT |
|  | *Brassica rapa* | 387 | + | CAAAT |
|  | *Glycine max* | 443 | + | CAATT |
|  | *Hordeum vulgare* | 510 | + | CAAT |
|  | *Brassica rapa* | 537 | + | CAAAT |
|  | *Glycine max* | 623 | - | CAATT |
|  | *Arabidopsis thaliana* | 624 | - | CCAAT |
|  | *Hordeum vulgare* | 697 | - | CAAT |
|  | *Glycine max* | 714 | + | CAATT |
|  | *Brassica rapa* | 796 | - | CAAAT |
| [CCAAT-box](http://bioinformatics.psb.ugent.be/webtools/plantcare/cgi-bin/show_site_info.htpl?QWhere=ID_of_Site like 'HV~CCAAT-box'&StartAt=0&NbRecs=10) - MYBHv1 binding site | *Hordeum vulgare* | 493 | - | CAACGG |
| [GA-motif](http://bioinformatics.psb.ugent.be/webtools/plantcare/cgi-bin/show_site_info.htpl?QWhere=ID_of_Site like 'GM~GA-motif'&StartAt=0&NbRecs=10) - part of a light responsive element | *Glycine max* | 363 | - | AAGGAAGA |
| [GCN4_motif](http://bioinformatics.psb.ugent.be/webtools/plantcare/cgi-bin/show_site_info.htpl?QWhere=ID_of_Site like 'OS~GCN4_motif'&StartAt=0&NbRecs=10) - cis-regulatory element involved in endosperm expression | *Oryza sativa* | 205 | + | CAAGCCA |
| [GT1-motif](http://bioinformatics.psb.ugent.be/webtools/plantcare/cgi-bin/show_site_info.htpl?QWhere=ID_of_Site like 'AS~GT1-motif'&StartAt=0&NbRecs=10) - light responsive element | *Avena sativa* | 627 | + | GGTTAAT |
| [Gap-box](http://bioinformatics.psb.ugent.be/webtools/plantcare/cgi-bin/show_site_info.htpl?QWhere=ID_of_Site like 'AT~Gap-box'&StartAt=0&NbRecs=10) - part of a light responsive element | *Arabidopsis thaliana* | 387 | + | CAAATGAA(A/G)A |
| [HSE](http://bioinformatics.psb.ugent.be/webtools/plantcare/cgi-bin/show_site_info.htpl?QWhere=ID_of_Site like 'BO~HSE'&StartAt=0&NbRecs=10) - cis-acting element involved in heat stress responsiveness | *Brassica oleracea* | 522 | - | AAAAAATTTC |
| [L-box](http://bioinformatics.psb.ugent.be/webtools/plantcare/cgi-bin/show_site_info.htpl?QWhere=ID_of_Site like 'LE~L-box'&StartAt=0&NbRecs=10) - part of a light responsive element | *Lycopersicon esculentum* | 624 | - | AAATTAACCAAC |
| [P-box](http://bioinformatics.psb.ugent.be/webtools/plantcare/cgi-bin/show_site_info.htpl?QWhere=ID_of_Site like 'OS~P-box'&StartAt=0&NbRecs=10) - gibberellin-responsive element | *Oryza sativa* | 101 | + | CCTTTTG |
| [Skn-1_motif](http://bioinformatics.psb.ugent.be/webtools/plantcare/cgi-bin/show_site_info.htpl?QWhere=ID_of_Site like 'OS~Skn-1_motif'&StartAt=0&NbRecs=10) - cis-acting regulatory element required for endosperm expression | *Oryza sativa* | 64 | + | GTCAT |
| [Sp1](http://bioinformatics.psb.ugent.be/webtools/plantcare/cgi-bin/show_site_info.htpl?QWhere=ID_of_Site like 'OS~Sp1'&StartAt=0&NbRecs=10) - light responsive element | *Oryza sativa* | 829 | + | GGGCGG |
| [TATA-box](http://bioinformatics.psb.ugent.be/webtools/plantcare/cgi-bin/show_site_info.htpl?QWhere=ID_of_Site like 'AT~TATA-box'&StartAt=0&NbRecs=10) - Core promoter element around -30 of transcription start | *Arabidopsis thaliana* | 117 | + | TAAAGATT |
|  | *Arabidopsis thaliana* | 167 | - | TATAAATATAAA |
|  | *Arabidopsis thaliana* | 168 | - | TATAA |
|  | *Arabidopsis thaliana* | 169 | + | TATA |
|  | *Arabidopsis thaliana* | 177 | + | TAAAGATT |
|  | *Arabidopsis thaliana* | 291 | - | TATAA |
|  | *Arabidopsis thaliana* | 292 | + | TATA |
|  | *Brassica oleracea* | 345 | + | ATATAA |
|  | *Arabidopsis thaliana* | 346 | + | TATAAA |
|  | *Arabidopsis thaliana* | 409 | - | ccTATAAAaa |
|  | *Lycopersicon esculentum* | 410 | + | TTTTA |
|  | *Arabidopsis thaliana* | 429 | + | TATA |
|  | *Glycine max* | 431 | - | TAATA |
|  | *Pisum sativum* | 480 | - | TATATGT |
|  | *Brassica napus* | 482 | + | ATATAT |
|  | *Arabidopsis thaliana* | 483 | + | TATA |
|  | *Arabidopsis thaliana* | 511 | - | tcTATATAtt |
|  | *Brassica napus* | 512 | + | ATATAT |
|  | *Arabidopsis thaliana* | 513 | + | TATATATA |
|  | *Brassica napus* | 514 | + | ATATAT |
|  | *Arabidopsis thaliana* | 515 | + | TATA |
|  | *Brassica oleracea* | 516 | + | ATATAA |
|  | *Arabidopsis thaliana* | 517 | + | TATA |
|  | *Pisum sativum* | 525 | - | TATAAAAT |
|  | *Arabidopsis thaliana* | 526 | - | TATAAAA |
|  | *Arabidopsis thaliana* | 527 | - | TATAAA |
|  | *Arabidopsis thaliana* | 528 | - | TATATAA |
|  | *Arabidopsis thaliana* | 529 | + | TATA |
|  | *Arabidopsis thaliana* | 531 | + | TATA |
|  | *Lycopersicon esculentum* | 544 | + | TTTTA |
|  | *Zea mays* | 586 | - | TTTAAAAA |
|  | *Lycopersicon esculentum* | 587 | + | TTTTA |
|  | *Zea mays* | 588 | + | TTTAAAAA |
|  | *Lycopersicon esculentum* | 590 | - | TTTTA |
|  | *Lycopersicon esculentum* | 609 | - | TTTTA |
|  | *Arabidopsis thaliana* | 620 | + | TATA |
|  | *Lycopersicon esculentum* | 633 | + | TTTTA |
|  | *Glycine max* | 636 | + | TAATA |
|  | *Lycopersicon esculentum* | 653 | + | TTTTA |
|  | *Arabidopsis thaliana* | 666 | - | TATTTAAA |
|  | *Glycine max* | 672 | + | TAATA |
|  | *Lycopersicon esculentum* | 675 | - | TTTTA |
|  | *Lycopersicon esculentum* | 707 | + | TTTTA |
|  | *Arabidopsis thaliana* | 726 | + | TAAAAATAA |
|  | *Lycopersicon esculentum* | 732 | - | TTTTA |
|  | *Glycine max* | 767 | + | TAATA |
|  | *Brassica oleracea* | 780 | + | ATATAAT |
|  | *Arabidopsis thaliana* | 781 | + | TATA |
|  | *Lycopersicon esculentum* | 856 | - | TTTTA |
| [TCA-element](http://bioinformatics.psb.ugent.be/webtools/plantcare/cgi-bin/show_site_info.htpl?QWhere=ID_of_Site like 'BO~TCA-element'&StartAt=0&NbRecs=10) - cis-acting element involved in salicylic acid responsiveness | *Brassica oleracea* | 100 | - | CAGAAAAGGA |
|  | *Brassica oleracea* | 498 | + | GAGAAGAATA |
| [Unnamed__4](http://bioinformatics.psb.ugent.be/webtools/plantcare/cgi-bin/show_site_info.htpl?QWhere=ID_of_Site like 'PH~Unnamed__4'&StartAt=0&NbRecs=10) | *Petroselinum hortense* | 13 | + | CTCC |
|  | *Petroselinum hortense* | 436 | - | CTCC |
|  | *Petroselinum hortense* | 56 | - | CTCC |
|  | *Petroselinum hortense* | 862 | + | CTCC |
| [Unnamed__6](http://bioinformatics.psb.ugent.be/webtools/plantcare/cgi-bin/show_site_info.htpl?QWhere=ID_of_Site like 'ZM~Unnamed__6'&StartAt=0&NbRecs=10) | *Zea mays* | 647 | - | taTAAATATct |
| [WUN-motif](http://bioinformatics.psb.ugent.be/webtools/plantcare/cgi-bin/show_site_info.htpl?QWhere=ID_of_Site like 'BO~WUN-motif'&StartAt=0&NbRecs=10) - wound-responsive element | *Brassica oleracea* | 113 | - | TCATTACGAA |
| [circadian](http://bioinformatics.psb.ugent.be/webtools/plantcare/cgi-bin/show_site_info.htpl?QWhere=ID_of_Site like 'LE~circadian'&StartAt=0&NbRecs=10) - cis-acting regulatory element involved in circadian control | *Lycopersicon esculentum* | 696 | - | CAANNNNATC |

**Table S4. Predicted regulatory domains of St*MKK6* native promoter from *S. tuberosum*, cv. Santé. The promoter sequence is 247 bp-long and analyzed with PlantCare software [36].**

| **Site name** | **Organism** | **Position** | **Strand** | **Sequence** |
| --- | --- | --- | --- | --- |
| Box I - light responsive element | *Pisum sativum* | 84 | + | TTTCAAA |
|  | *Pisum sativum* | 212 | + | TTTCAAA |
|  | *Pisum sativum* | 173 | - | TTTCAAA |
|  | *Pisum sativum* | 240 | + | TTTCAAA |
| CAAT-box - common cis-acting element in promoter and enhance | *Hordeum vulgare* | 63 | - | CAAT |
|  | *Brassica rapa* | 162 | - | CAAAT |
|  | *Glycine max* | 80 | + | CAATT |
| Sp1 - light responsive element | *Oryza sativa* | 195 | + | GGGCGG |
| TATA-box - core promoter element around -30 of transcription start | *Arabidopsis thaliana* | 1 | + | TATA |
|  | *Glycine max* | 133 | + | TAATA |
|  | *Arabidopsis thaliana* | 92 | + | TAAAAATAA |
|  | *Arabidopsis thaliana* | 147 | + | TATA |
|  | *Lycopersicon esculentum* | 41 | - | TTTTA |
|  | *Brassica rapa* | 146 | + | ATATAAT |
|  | *Lycopersicon esculentum* | 98 | - | TTTTA |
|  | *Lycopersicon esculentum* | 222 | - | TTTTA |
|  | *Glycine max* | 38 | + | TAATA |
|  | *Lycopersicon esculentum* | 73 | + | TTTTA |
| Unnamed__3 | *Zea mays* | 12 | + | CGTGG |
| Unnamed__4 | *Petroselinum hortense* | 228 | + | CTCC |
| circadian | *Lycopersicon esculentum* | 62 | - | CAANNNNATC |

**Table S5**. **Expression values of *StMKK6* in control and SA-treated potatoes cv. Rywal and NahG-Rywal.** Two biological replicates per treatment were analysed. Relative expression values and fold-changes (compared to Rywal control) are shown in the table. Differences between control and SA-treated plants were statistically evaluated by t-test.

| **Rywal** | | **NahG-Rywal** | |
| --- | --- | --- | --- |
| control | SA | control | SA |
| 0.60 | 0.33 | 0.22 | 0.68 |
| 0.50 | 0.22 | 0.30 | 0.83 |
| Average: 0.55 | Average: 0.27 | Average: 0.23 | Average: 0.76 |
| 1 | 0.49 | 0.47 | 1.38 |
| t-test: 0.066 | | t-test: 0.027 | |

**Table S6. Comparison of *StMKK6* basal expression values in Rywal and NahG-Rywal plants.** Two biological replicates per treatment were analysed. Relative expression values and fold-changes (compared to Rywal control) are shown in the table. Differences between Rywal and NahG-Rywal plants were statistically evaluated by t-test.

| **Rywal** | **NahG-Rywal** |
| --- | --- |
| 0.60 | 0.22 |
| 0.50 | 0.30 |
| Average: 0.55 | Average: 0.23 |
| 1 | 0,49 |
| t-test: 0.045 | |

**Table S7. Subcellular localisation prediction of StMKK6 and AtMKK6.** Amino acid sequence of AtMKK6 (GenBank accession number NM_125041.2) and StMKK6 (GenBank accession number KF837129.1) proteins were used as query in PredictProtein service. The localisation for each was predicted by three different prediction algorithms: PROSITE, LOCkey and LOCtree. For each subcellular prediction a confidence to the prediction is given.

| **Localisation prediction algorithm** | **Protein** | **Predicted localization** | **Confidence** |
| --- | --- | --- | --- |
| PROSITE | StMKK6 | Nuclear | 84 |
|  | AtMKK6 | Nuclear | 84 |
| LOCkey | StMKK6 | Cytoplasmic | 100 |
|  | AtMKK6 | Cytoplasmic | 100 |
| LOCtree | StMKK6 | Chloroplast | 2 |
|  | AtMKK6 | Chloroplast | 3 |
